# High altitude is associated with pTau deposition, neuroinflammation, and myelin loss

**DOI:** 10.1038/s41598-022-10881-x

**Published:** 2022-04-27

**Authors:** Diego Iacono, Erin K. Murphy, Paul M. Sherman, Holly Chapapas, Bianca Cerqueira, Christine Christensen, Daniel P. Perl, John Sladky

**Affiliations:** 1 DoD/USU Brain Tissue Repository & Neuropathology Program, Uniformed Services University (USU), Bethesda, MD USA; 2 Department of Neurology, F. Edward Hébert School of Medicine, Uniformed Services University (USU), Bethesda, MD USA; 3 Department of Pathology, F. Edward Hébert School of Medicine, Uniformed Services University (USU), Bethesda, MD USA; 4 Neuroscience Program, Department of Anatomy, Physiology and Genetics, F. Edward Hébert School of Medicine, Uniformed Services University (USU), Bethesda, MD USA; 5 The Henry M. Jackson Foundation for the Advancement of Military Medicine (HJF), Inc., 6720B Rockledge Dr, Bethesda, MD 20817 USA; 6 Neurodegeneration Disorders Clinic, National Institute of Neurological Disorders and Stroke, NINDS, NIH, Bethesda, MD USA; 7 Department of Radiology, 59th Medical Wing, Joint Base San Antonio-Lackland (JBSA), San Antonio, TX USA; 8 Department of Radiology, F. Edward Hébert School of Medicine, Uniformed Services University (USU), Bethesda, MD USA; 9 Aeromedical Neurology & Neuroimaging Research Group, United States Air Force School of Aerospace Medicine, Joint Base San Antonio-Lackland (JBSA), San Antonio, TX USA; 10 KBR Science & Space, San Antonio, TX USA; 11 U.S. Army Veterinary Corps, 59th Clinical Investigation and Research Support, JBSA-Lackland, San Antonio, TX USA; 12 Department of Neurology, Brooke Army Medical Center, Ft. Sam Houston, TX USA

**Keywords:** Neuroscience, Cellular neuroscience

## Abstract

Mammals are able to adapt to high altitude (HA) if appropriate acclimation occurs. However, specific occupations (professional climbers, pilots, astronauts and other) can be exposed to HA without acclimation and be at a higher risk of brain consequences. In particular, US Air Force U2-pilots have been shown to develop white matter hyperintensities (WMH) on MRI. Whether WMH are due to hypoxia or hypobaria effects is not understood. We compared swine brains exposed to 5000 feet (1524 m) above sea level (SL) with 21% fraction inspired O_2_ (FiO_2_) (Control group [C]; n = 5) vs. 30,000 feet (9144 m) above SL with 100% FiO_2_ group (hypobaric group [HYPOBAR]; n = 6). We performed neuropathologic assessments, molecular analyses, immunohistochemistry (IHC), Western Blotting (WB), and stereology analyses to detect differences between HYPOBAR vs. Controls. Increased neuronal insoluble hyperphosphorylated-Tau (pTau) accumulation was observed across different brain regions, at histological level, in the HYPOBAR vs. Controls. Stereology-based cell counting demonstrated a significant difference (*p* < 0.01) in pTau positive neurons between HYPOBAR and C in the Hippocampus. Higher levels of soluble pTau in the Hippocampus of HYPOBAR vs. Controls were also detected by WB analyses. Additionally, WB demonstrated an increase of IBA-1 in the Cerebellum and a decrease of myelin basic protein (MBP) in the Hippocampus and Cerebellum of HYPOBAR vs. Controls. These findings illustrate, for the first time, changes occurring in large mammalian brains after exposure to nonhypoxic-hypobaria and open new pathophysiological views on the interaction among hypobaria, pTau accumulation, neuroinflammation, and myelination in large mammals exposed to HA.

## Introduction

For different reasons, human and animal populations have spread and adapted to live at different elevations above sea level (SL). SL is the geo-physical point of reference for all chemical, physical, physiological, and metabolic parameters normally measured in the context of medical and biological human sciences. However, humans can be exposed to high altitude (HA), defined as an altitude of ≥ 8202 feet (2500 m) above SL, for different leisure or occupational purposes^1^.

HA-exposed subjects may experience significant clinical phenomena due to the mutated physico-environmental conditions generated by barometric and partial pressure of inspired oxygen (PaO_2_) alterations^2^. Pilots, aviators, miners, astronauts, military, altitude chamber personnel, climbers, alpinists, researchers (i.e. astronomers, anthropologists, medical investigators, etc.) and others, can be all potentially affected by the detrimental effects of HA, especially when exposed to HA over a short period of time and without procedures of acclimatization and prophylaxis^3–5^. Generally, until certain physiological limits are exceeded, systematic and gradual periods of acclimatization permit most living organisms to activate and stabilize a series of physiological and biochemical adaptive mechanisms^3–6^. These adaptive mechanisms allow body systems, organs and corresponding functions (lung/respiration, blood hemoglobin/blood oxygenation, vasculature reactivity/blood pressure, liver/glucose metabolism, central nervous system/consciousness-cognition-behaviour-movement, and other) to satisfactorily adapt to the new mutated environmental conditions^5^.

However, HA-related over-threshold physico-chemical-environmental changes, either acute or chronic, may induce severe physiological and metabolic alterations that can culminate into manifestations of different clinical syndromes. These syndromes are signals of the abnormal functioning of different organs and systems, including the central nervous system (CNS), which are particularly vulnerable to the lowered levels of arterial blood oxygenation and blood pressure changes^7,8^. In particular, acute hypoxic–hypobaric conditions such as those generated by rapid ascension to HA may be associated with a wide spectrum of clinical phenomena usually referred to as acute mountain sickness (AMS), high-altitude sickness (HAS)^9,10^, decompression sickness (DCS)^11^ or neurological decompression sickness (NDCS), when neurological signs and symptoms are predominant^12,13^. By contrast, chronic exposure to HA may be associated with other different types of clinical manifestations such as Monge's disease or chronic mountain sickness (CMS), which is a characteristic maladaptive condition caused by chronic (years) exposure to HA^14–16^.

Studies enrolling US Air Force (USAF) U2-pilots, which are military pilots receiving intense periods of training and who operate at an altitude of ≥ 30,000 feet (≥ 9144 m) above SL, have reported brain abnormalities characterized by diffuse subcortical white matter (WM) hyperintensities (WMH) detected by T2-weighted MRI sequence protocols in this specific military cohort^17,18^. Remarkably, WMH have been identified in U2-pilots despite the fact that this highly specialized military population operates under sophisticated controlled-pressurized systems while successfully performing exceptionally demanding missions and operations. These WMH lesions have been more often observed prior to the implementation of the CARE (Cockpit Altitude Reduction Effort) program in 2012–2013, which increased cabin pressurization from 4.4 psi (29,500 feet/8991.6 m equivalent) to 7.65 psi (15,000 feet/4572 m equivalent). However, these brain alterations are still observed. WMH are not unique to U2-pilots though. In fact, WMH have been described in other military populations such as personnel exposed to altitude chambers and in non-military populations as well^19–22^. HA-related WMH lesions have been hypothesized to be mainly produced by the detrimental effects of hypoxia-hypobaria conditions that can be present at HA, especially when extreme high altitudes (ex-HAs)—altitudes  ≥ 90,000 feet/27,432 m above SL—have been reached. Military pilots can be exposed to ex-HAs and, in these situations, mandatory pre-flight mitigating protocols are required (e.g. nitrogen degassing and other)^23^.

Specifically, WMH in U2-pilots have been described as hyperintense MRI signals of differing size and volume and anatomically distributed across the entire thickness of subcortical myelinated brain regions^18^. WMH were detectable using specific fluid-attenuated inversion recovery (FLAIR) T2-weighted MRI protocols^24^. Interestingly, WMH in U2-pilots did not show linear correlations with total number of cumulative hours of nonhypoxic–hypobaric exposure^24^. Furthermore, U2-pilots with high burdens of WMH had poorer reasoning/calculation, memory, information processing accuracy, general cognitive proficiency, and general cognitive processing MicroCog scores when compared to U2-pilots with low WMH burden^24^. Notably, regardless of the statistical differences in test performance, U2-pilots continue to be on par with age and cohort specific normative data, indicating that the differences in neurocognitive performance are not of immediate clinical significance. However, when comparing U2 pilots to USAF pilots flying other platforms, U2 pilots had lower reasoning/calculation, memory, information processing accuracy, and general cognitive functioning MicroCog scores^25^.

WMH lesions and related cognitive disorders have been explained as due to the cumulative effects of hypoxic-related mechanisms commonly associated with HA and hypobaria in particular. As postulated by the “*bubbling theory*”^26^, hypobaria induces the formation of gaseous microbubbles (< 30 µm of diameter) and consequently, alterations of blood-gas alveolar exchanges and corresponding metabolic sequelae. The bubbling theory postulates that hypobaria generates gaseous microbubbles that function as the main, if not the only, causative factor of the arteriolar damage subsequent to HA exposure. While hypothesized to be linked to the gaseous bubbling phenomena associated with hypoxic-hypobaric or nonhypoxic-hypobaric conditions, the specific neuropathological nature of WMH in HA-exposed subjects has not been fully clarified or systematically assessed using post-mortem human or larger mammalian brains such as swine brains, for example. Furthermore, other HA-related pathomechanisms including neuroinflammatory response, immune system activation, blood–brain-barrier permeability changes, and protein expression level abnormalities (i.e., altered levels of total-tau, phosphorylated-tau, amyloid precursor protein (APP), *β*-amyloid or other proteins associated with cognitive disorders such as mild cognitive impairment [MCI], Alzheimer’s disease [AD], behavioral variant frontotemporal dementia [bvFTD] or other neurodegenerative conditions)^27^, cannot be completely excluded as additional or possible long-term effects of HA and hypobaria in particular.

While a series of animal studies described some neuropathological, neurochemical and cognitive changes due to HA exposure, most of these studies were performed in rodents^28–33^. Using large mammalian brains, such as swine brains, whose general metabolism and physiology are far more similar to humans than rodents, would offer numerous advantages into identifying possible specific pathophysiological, biochemical, imaging and pathological alterations of the brain due to HA, and specifically to hypobaria. The use of swine (minipigs) for HA studies has been already proposed and proven to be a very valuable tool of research for HA studies^34^. However, a systematic neuropathological and molecular evaluation assessing large mammalian brains experimentally-exposed to nonhypoxic-normobaric vs. nonhypoxic-hypobaric conditions has never been attempted before. The main rationale for this initial neuropathological investigation consisted in the possibility to identify specific types of histopathological lesions and molecular alterations in the brain of large mammals experimentally exposed to nonhypoxic-normobaric vs. nonhypoxic-hypobaric conditions. To notice, the possibility to experimentally create and control hypobaric conditions in the absence of hypoxic changes is not ordinarily achievable, especially in humans. Consequently, the possibility to assess brains of larger mammals, such as swine, specifically exposed to hypobaria represented a distinctive chance to provide some major advancements in the HA research field. Moreover, the identification of specific hypobaria-related brain effects could imply enormous repercussions in the future, especially in terms of ideation and production of more effective anti-hypobaria preventive tools and mitigation strategies to apply to either military and civilian populations exposed for occupational or leisure reasons to HA or ex-HAs.

We had indeed the unique opportunity to assess a series of swine brains exposed to different oxemic and barometric conditions in order to identify possible specific neuropathological lesions, cerebral tissue alterations and molecular changes present across different regions of the cerebrum, cerebellum, and brainstem. We assessed different components of the neural tissue (white matter [WM], gray matter [GM], cerebral vessels, neurons, astroglial and microglial cells) in nonhypoxic-hypobaric (hypobaric group [HYPOBAR]) vs. age- and sex-matched swine exposed to nonhypoxic-normobaric (control group [C]) conditions. In addition, by Western Blotting (WB) analyses, we performed quantitative protein expression level measurements of the following molecules: total-Tau (HT7), two forms of phosphorylated-Tau (pTau; S202 and AT8); glial fibrillary acidic protein (GFAP); ionized calcium binding adaptor molecule 1 (IBA1); myelin basic protein (MBP); and amyloid precursor protein (APP), in the Cortex [Cortex], Hippocampus [Hippo], Cerebellum [CRB] and Brainstem [BS] in both animal groups to detect possible significant differences between HYPOBAR and C.

Furthermore, we used stereological tools to quantify and compare the number of lesions (i.e. AT8-positive cells) found in HYPOBAR vs. C.

## Results

### General histology assessment (HE, CV, LFB-PAS)

The histological assessments based on H&E stain across all examined cerebral, cerebellar, and brainstem regions (fronto-, orbito-, temporal-, parietal- cortex, hippocampus, amygdala, striatum, occipital cortex, cerebellar cortex, and brainstem) did not show any vascular lesions (macro- or micro-hemorrhages, necrotic-ischemic lesions, perivascular space enlargement) in either HYPOBAR or C brains (Supplementary Fig. 1). CV stain showed no differences in terms of cytoarchitectural structures, neuronal histological distribution across cortices or cortical neuronal layers, and nuclear-cytoplasm ratios between HYPOBAR and C. No cytoplasmic, nuclear, or nucleolar morphological alterations were detectable across all examined regions and animal groups (Supplementary Fig. 2).

**Figure 1:**
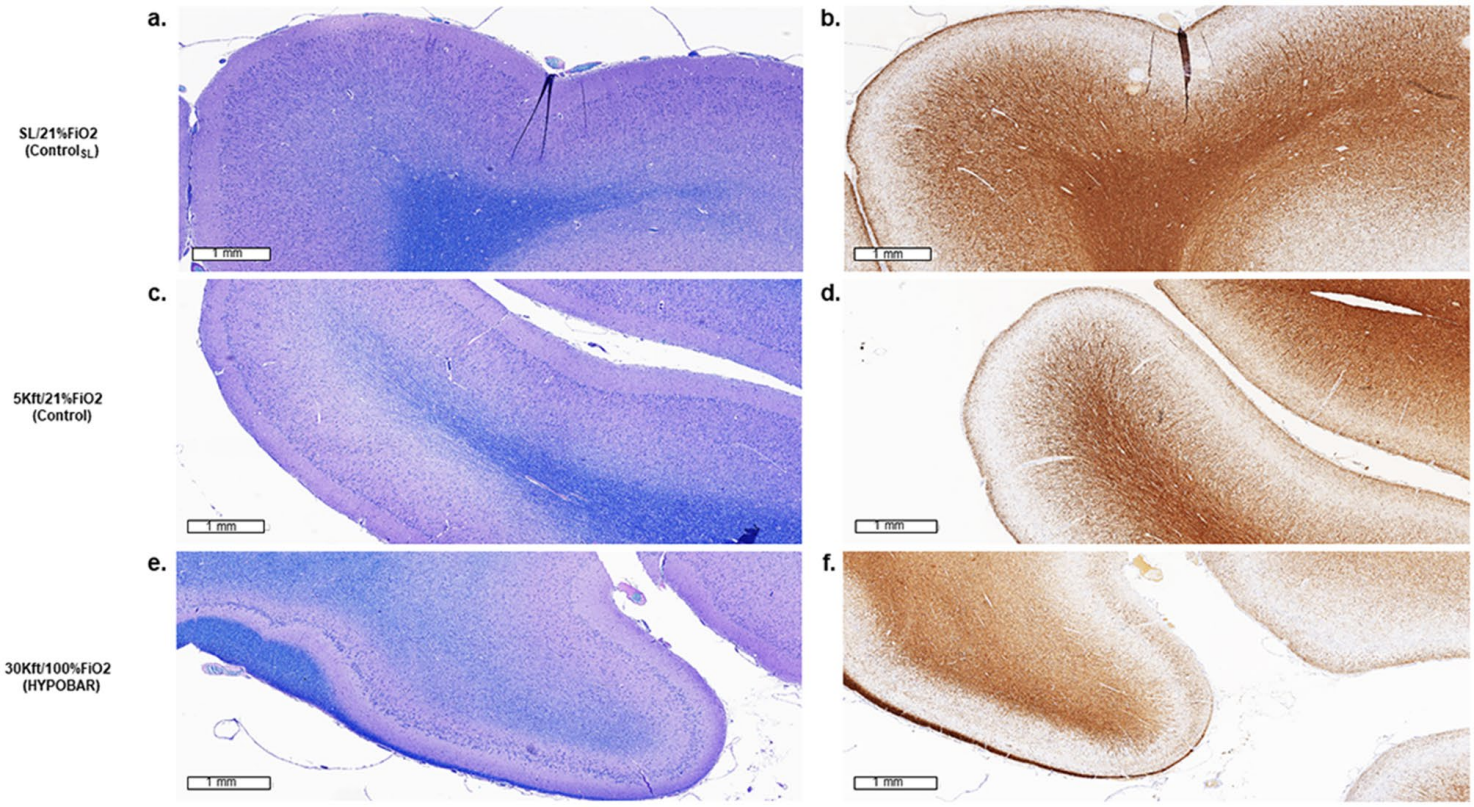
Luxol-fast Blue (LFB) stain (**a**,**c**,**e**) and immunohistochemistry (IHC) outcomes of MBP (myelin basic protein) antibody (**b**,**d**,**f**) in the left parietal cortex of minipig brains exposed to different oxemic and barometric conditions. All panels are at a scale of 1 mm (mm). Note the marked level of myelin rarefaction (**e**,**f**) in the cortical white matter of HYPOBAR vs. the other two experimental groups [CSL (**a**,**b**) and C (**c**,**d**) group]. *SL* sea level, *FiO*_*2*_ fraction inspired oxygen, *HYPOBAR* nonhypoxic-hypobaric condition, *ft* feet, *ControlSL* SL/21%FiO_2_, *Control* 5Kft/21%FiO_2_.

LFB-PAS stain showed diffuse myelin rarefaction without eosinophilic proteinaceous parenchymal depositions (as per PAS reaction) in HYPOBAR vs. C. The myelin rarefaction was observed across almost all cortical regions, especially at level of the WM subjacent the cortical gyri, see for example parietal cortex of HYPOBAR vs. C animals (Fig. 1, panel a and c vs. e). Intriguingly, the myelin rarefaction appeared to be homogeneously diffuse across WM areas without for example, preferentially affecting or preserving myelinated U-fibers^35^.

### Immunohistochemistry findings

**Figure 2:**
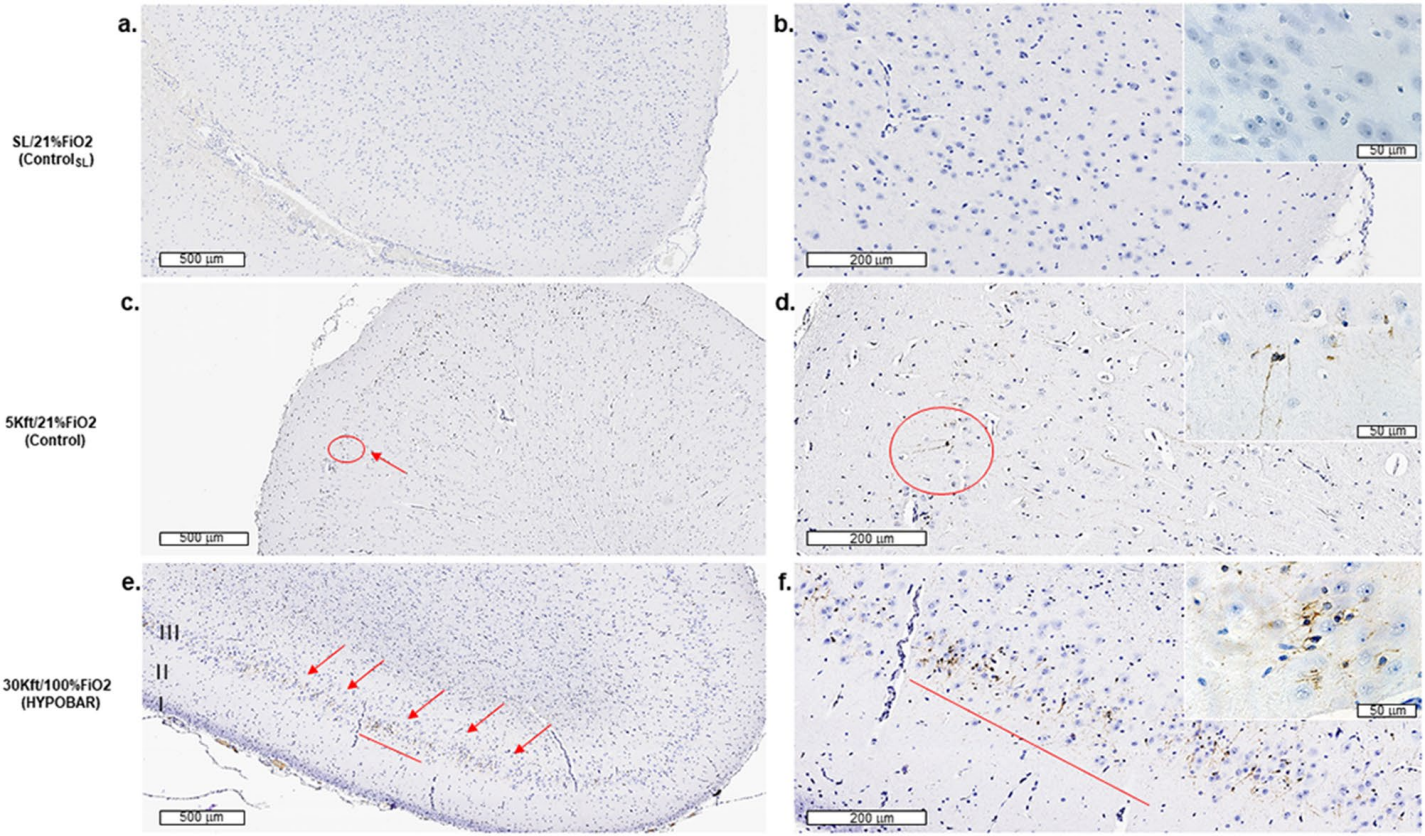
Immunohistochemistry (IHC) outcomes of the AT8 antibody in the left parietal cortex of minipig brains exposed to different oxemic and barometric conditions. Panel (**a**–**f**) show sections of the left parietal cortex at a lower level of magnification [at a scale of 500 microns (µm)] across all three analyzed experimental conditions. Panel (**b**), (**d**), (**f**) include upper right insets at a higher level of magnification [at a scale of 50 microns (µm)]. I, II, III indicate cortical layers. Red signals indicate the presence of AT8 positive cortical cells. Note the higher level of AT8-positive cortical cells in the 30ft/100%FiO_2_ (HYPOBAR) group. *SL* sea level, *FiO*_*2*_ fraction inspired oxygen, *HYPOBAR* nonhypoxic-hypobaric condition, *ft* feet, *ControlSL* SL/21%FiO_2_, *Control* 5Kft/21%FiO_2_.

*pTau*. S202 (CP13) and AT8 immunostain showed increased positive immunoreactivity (IR) across almost all cerebro-cortical (fronto-, parietal-, hippocampal cortex) regions examined in HYPOBAR vs. C. Specifically, small neuronal cells located at the level of layer III of the swine cerebral cortex were positive for intraneuronal AT8 accumulation (Fig. 2, panels a and c vs. e; panels b and d vs. f). Sparse AT8 positivity was also found in neuronal dendrites and axons. Low-magnification (20×) assessment showed possible differences between HYPOBAR and C in terms of region-based anatomical distribution and AT8 positivity loads. In fact, analyzing all cortical areas along the rostro-caudal direction of the brain (longitudinal fronto-occipital axis of the cerebral hemisphere), AT8 lesions were more frequent in the temporo-parietal region of the cortex and dentate gyrus (DG) of the Hippo in HYPOBAR vs. C. No AT8 positive neurons or other neural cells were detected in CRB and BS regions of either HYPOBAR or C animals. In the C_SL_ group, AT8 immunostains were negative across all brain regions analyzed.

**Figure 3:**
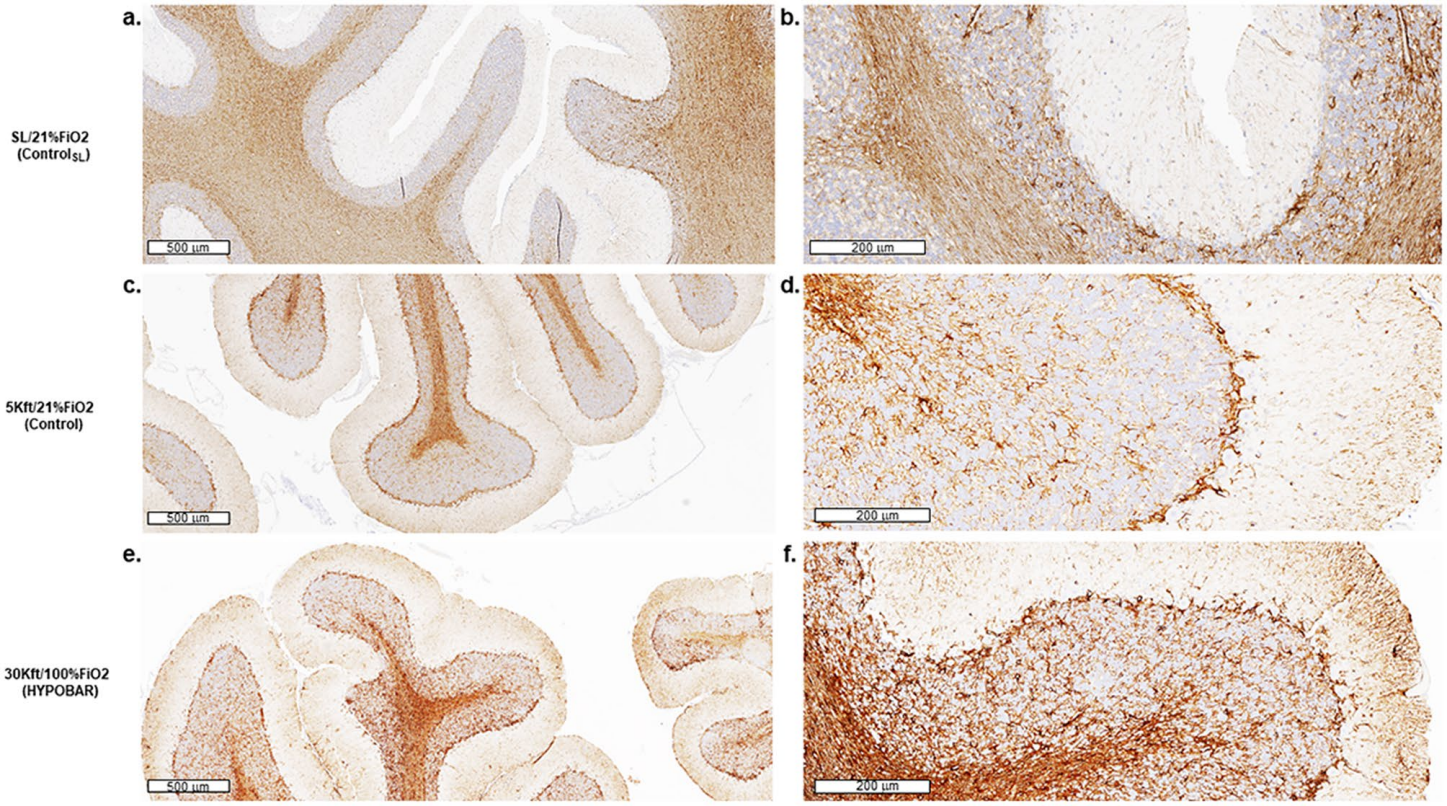
Immunohistochemistry (IHC) outcomes of the GFAP antibody in the left cerebellar hemisphere of minipig brains exposed to different oxemic and barometric conditions. Panel (**a**), (**c**), (**e**) show sections of the left cerebellar cortex at a lower level of magnification [at a scale of 600 microns (µm)] across all three analyzed conditions. Panels (**b**), (**d**), (**f**) show the same regions at a higher level of magnification [at a scale of 200 microns (µm)]. Note the higher astroglial response in the cerebellar “arbor vitae” of HYPOBAR vs. the other two groups. *SL* sea level, *FiO*_*2*_ fraction inspired oxygen, *HYPOBAR* nonhypoxic-hypobaric condition, *ft* feet, *ControlSL* SL/21%FiO_2_, *Control* 5Kft/21%FiO_2_.

*GFAP.* IR for GFAP was present across all examined cortical and subcortical brain regions analyzed across all groups. By light-microscopy at low magnification, astroglial cells appeared to be homogeneously located across the various brain regions without a region-specific astroglial reactivity, frank astrogliosis or astroglial scaring phenomena. However, light-microscopy inspection at high-magnification (40×) revealed diffuse nuclear hypertrophy in some astroglial cells across most of the examined brain regions, especially in cerebellar folia at level of the cerebellar WM and on the border between the molecular and Purkinje/granular layers (Fig. 3, panel a and c vs. e). More specifically, the microscopic qualitative-based comparison among groups showed a gradual increase of GFAP-based neuroinflammatory response across C_SL_ > 5 K/FiO_2_-21% > 30 K/FiO_2_-100% group (Fig. 3, panel b and d vs. f).

**Figure 4:**
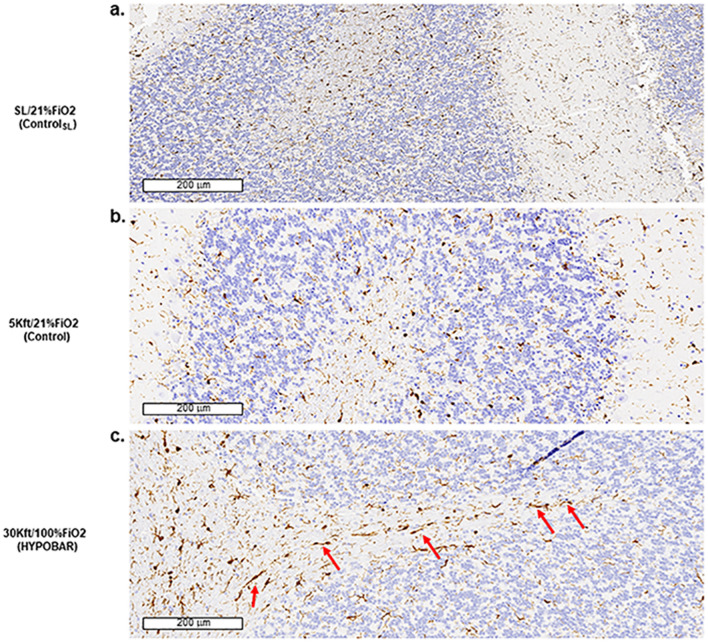
Immunohistochemistry (IHC) outcomes of IBA1 antibody. Panels (**a**–**c**) are at a scale of 200 microns (µm). Note in panel c the presence of rod-shape microglia cells in the cerebellar “arbor vitae” in the 30Kft/100%FiO_2_ (HYPOBAR) group in comparison to the other two groups (SL/21%FiO_2_ and 5Kft/21%FiO_2_). *SL* sea level, *FiO*_*2*_ fraction inspired oxygen, *HYPOBAR* nonhypoxic-hypobaric condition, *ft* feet, *ControlSL* SL/21%FiO_2_, *Control* 5Kft/21%FiO_2_.

*IBA-1.* IR for IBA-1 positive microglia cells was present across all anatomical regions and subregions examined in all animal groups. However, a suggestion of microglial activation, as detected by thickening of the microglia cell bodies and ramifications was noticed in the Hippo and CRB of HYPOBAR vs. C. However, it was not possible to semi-quantitatively separate the degree of activated microglial cells between HYPOBAR vs. C. Of particular interest was the rod-shape aspect of most microglial cells in the WM of cerebellar folia in the HYPOBAR group (Fig. 4, panel a and b vs. c).

*MBP.* IR for MBP showed reduced levels of myelination intensity across almost all cortical regions, especially at level of the WM subjacent cortical gyri (Fig. 1, panel b and d vs. f). Using contiguous tissue sections stained with LFB-PAS, IR for MBP confirmed the apparent reduction of myelination intensity.

*APP.* No extracellular or abnormal accumulation of insoluble APP-positive lesions in cortical or subcortical neurons or axons were detected in either HYPOBAR or C group.

### Immunofluorescence findings

**Figure 5:**
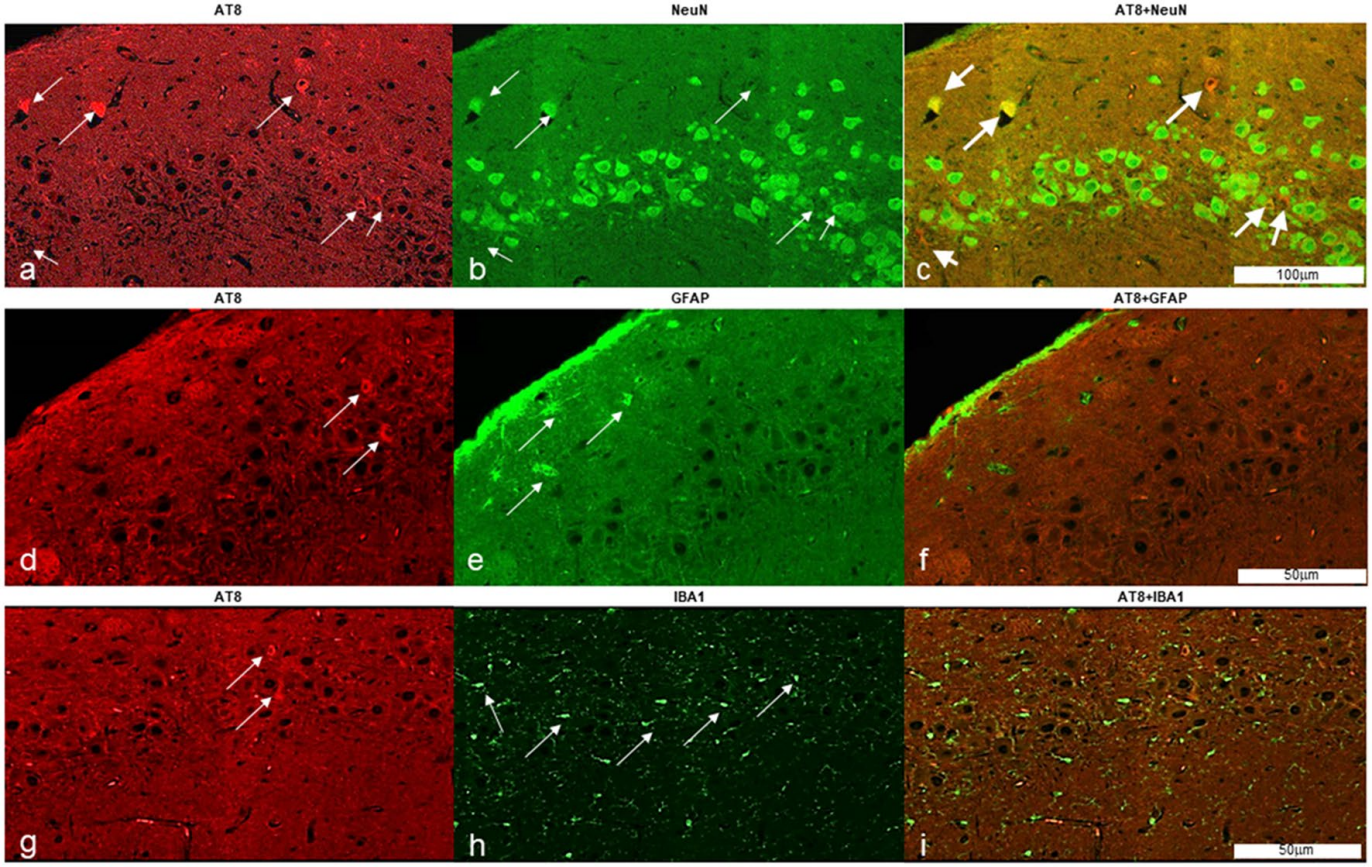
Hyperphosphorylated-Tau (pTau) accumulates in neurons after Hypobaric Exposure. Figure shows immunofluorescence reactivity for AT8 in hippocampal neurons of a HYPOBAR animal (**a**–**c**). AT8 did not colocalize with either astroglial (**d**–**f**) or microglial cells (**g**–**i**). Thin white arrows show AT8+, NeuN+, GFAP+ and IBA1+ positive cells in the hippocampal cortex of a HYPOBAR animal. Notice the specific accumulation of pTau (AT8) in neurons (Thick white arrows, **c**).

The application of immunofluorescence (IF) methods were able to show co-localization of AT8 deposition in cortical neurons of different brain regions (i.e. DG) in contrast to the absence of co-localization of AT8 deposition in either astroglial (GFAP stain) or microglial cells (IBA1 stain) (Fig. 5).

### Stereology findings

**Figure 6:**
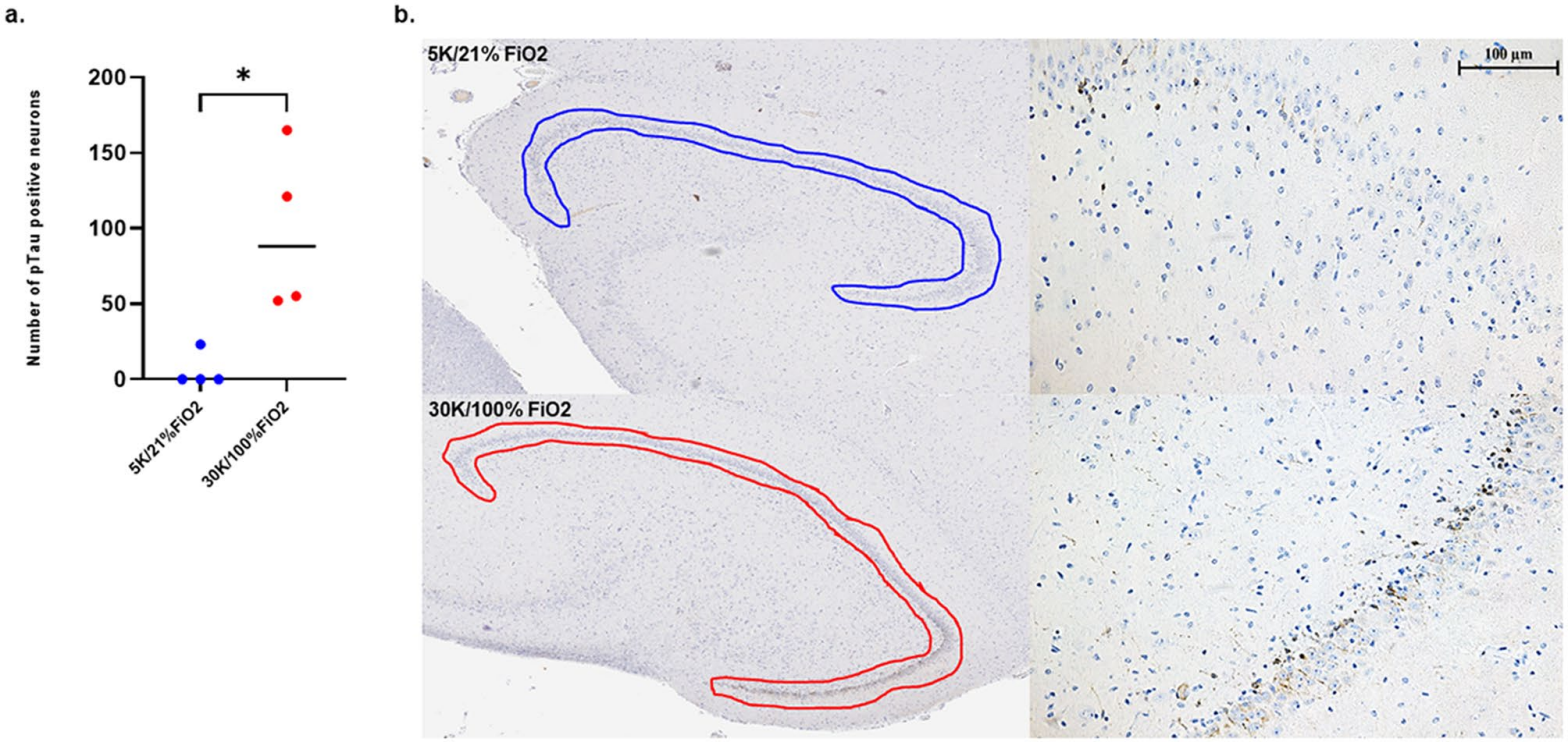
Stereological Counts for AT8 positive neurons in the Dentate Gyrus (DG) of the left Hippocampus. (**a**) Histogram representing the neuronal counting of AT8 positive neurons measured in the DG region of HYPOBAR (red spot) vs. C (blue spots) of a total of 4 animals for each experimental condition. (**b**) Histological images and contours of the region of interest analyzed for the stereology-based counting assessment. Of note are the relative higher frequency of AT8 positive neurons along the granular layer of the DG. *DG* dentate gyrus of the hippocampus.

Following stereology-based methods, a significantly higher number of AT8-positive neurons were counted in the DG of HYPOBAR group compared to C (*p* < 0.01) (Fig. 6).

### Western blotting quantification analyses

#### pTau

**Figure 7:**
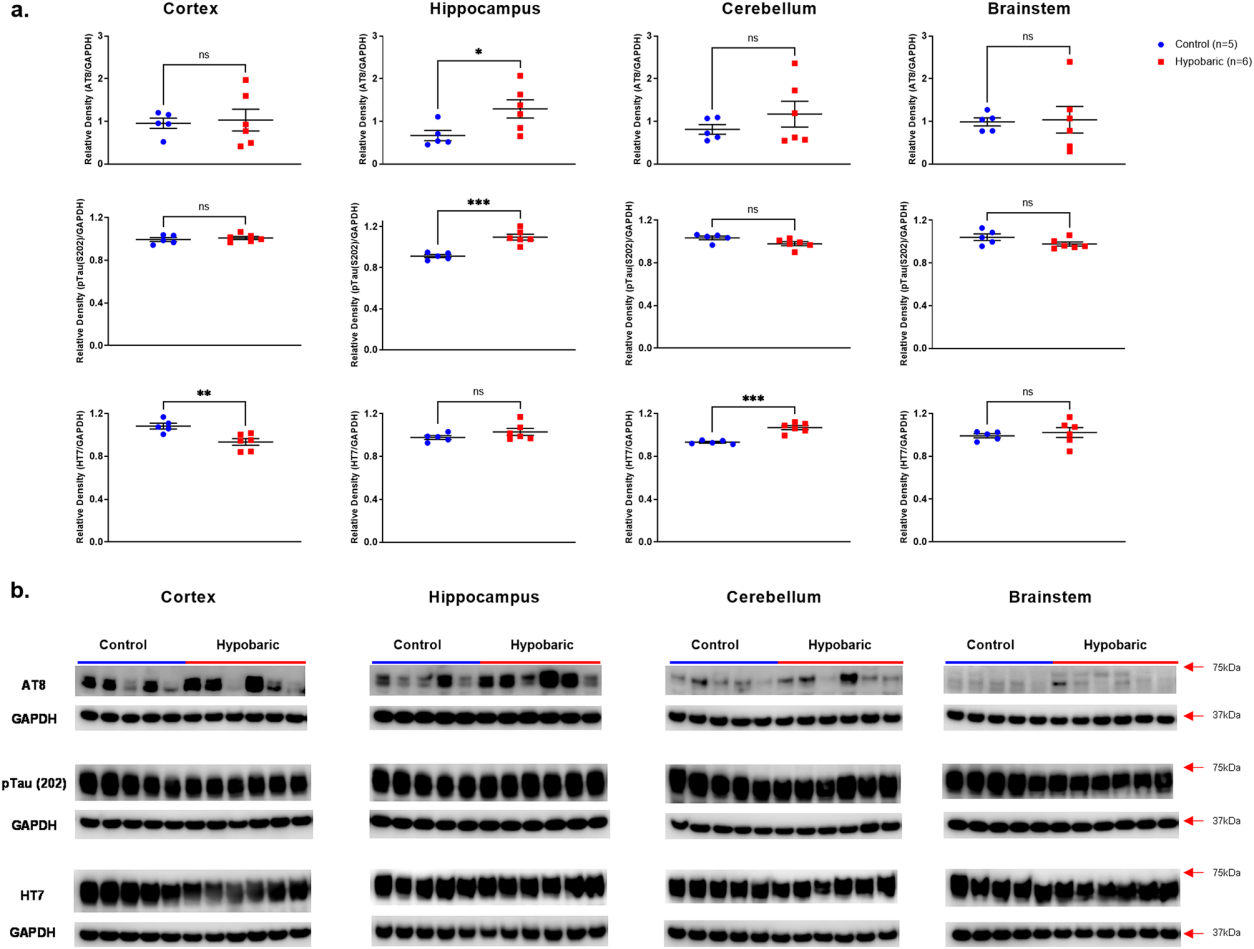
Phosphorylated and Total Tau Protein Expression following Hypobaric Exposure. (**a**) Histograms representing the densitometric ratio of levels of pTau-AT8 (Ser202 and Thr205), pTau (Ser202) and HT7 with respect to GAPDH in the Control (blue spots) vs. HYPOBAR (red squares) group as measured in the frontal cortex, hippocampus, cerebellum and brainstem. All gels were run in duplicate and data represents the average of 2 runs per sample. Error bars represent standard error of the mean (SEM). *p < 0.05; **p < 0.01 (two-tailed unpaired *t*-test). (**b**) Representative western blots for antibodies used. Full length blots are shown in Supplementary Figs. 3–9.

Confirming the identification of increased pTau at the histological level, significantly greater protein expression for both pTau antibodies, S202 (p = 0.0004) and AT8 (p = 0.04) were demonstrated in the Hippo of HYPOBAR vs. C. No differences in pTau expression levels were noted in Cortex, CRB or BS (Fig. 7).

#### Total-Tau (HT7)

Interestingly, there was some fluctuation in total tau levels as measured by HT7 expression levels. However, a significantly lower level of HT7 was demonstrated in the Cortex (p = 0.006) and a significantly greater level of HT7 was detected in the CRB (p = 0.0001) of HYPOBAR vs. C (Fig. 7). No changes in HT7 expression levels in HYPOBAR vs. C were seen in the Hippo or BS.

#### GFAP

**Figure 8:**
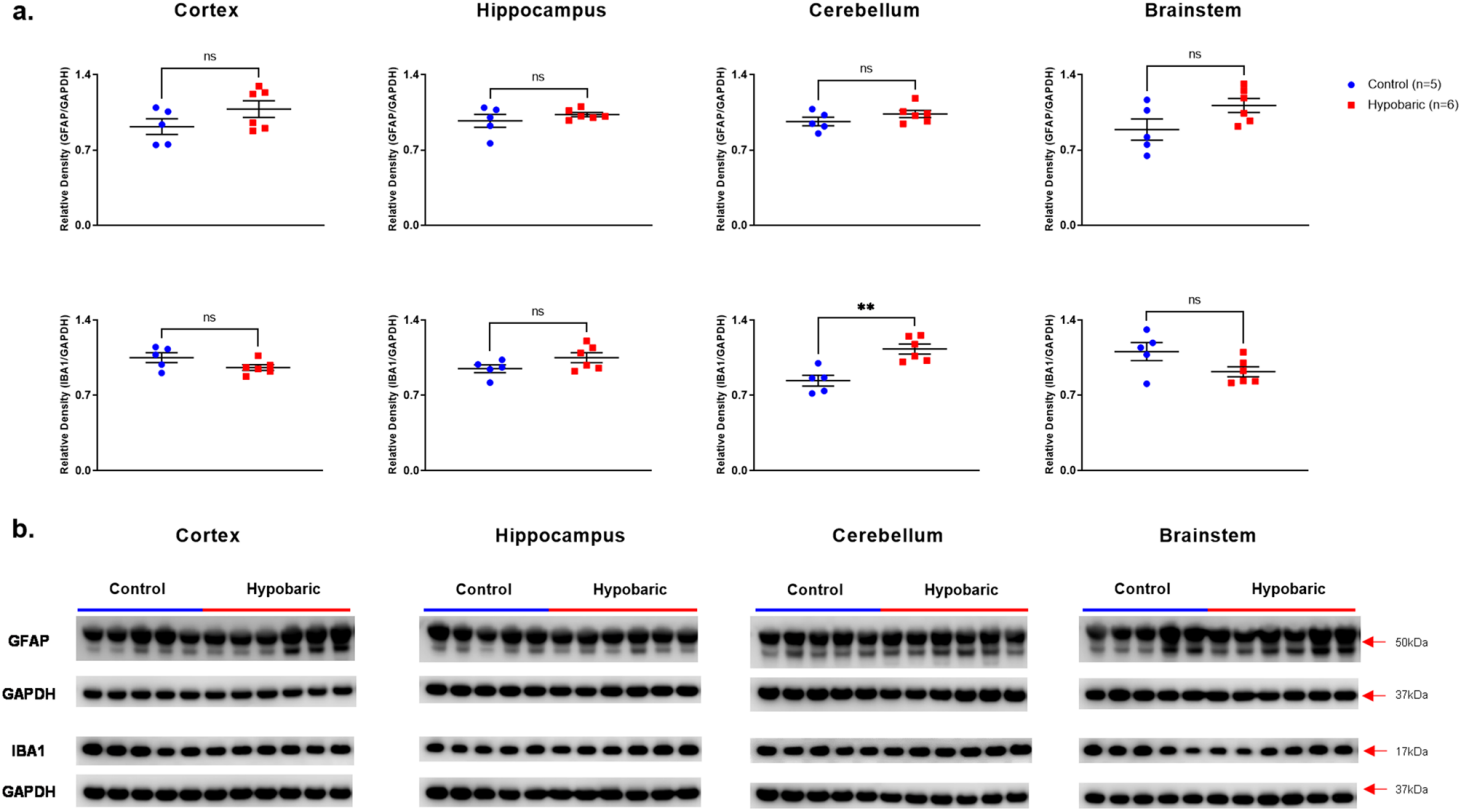
GFAP and IBA-1 Protein Expression Following Hypobaric Exposure. (**a**) Histograms representing the densitometric ratio of levels of GFAP and IBA1 with respect to GAPDH in the Control (blue spots) vs. HYPOBAR (red squares) group as measured in the frontal cortex, hippocampus, cerebellum and brainstem. All gels were run in duplicate and data represents the average of 2 runs per sample. Error bars represent standard error of the mean (SEM). *p < 0.05; **p < 0.01 (two-tailed unpaired *t*-test). (**b**) Representative western blots for antibodies used. Full length blots are shown in Supplementary Figs. 3–9.

Similarly to the immunohistochemistry observations for insoluble GFAP stain, slightly higher levels of soluble GFAP were detected across all brain regions (Cortex, Hippo, CRB, BS) of HYPOBAR vs. C. However, none of those differences reached the level of statistical significance (Fig. 8).

#### IBA-1

Higher levels of IBA-1 were detected in the Hippo and CRB of HYPOBAR vs. C, with statistical significance only in the CRB (p = 0.002). Again, this increase agreed with the IHC results for IBA1 in the Hippo and CRB. By contrast, lower levels of IBA-1 were detected in the Cortex and BS. These latter differences, however, did not reach statistical significance (Fig. 8).

#### APP

**Figure 9:**
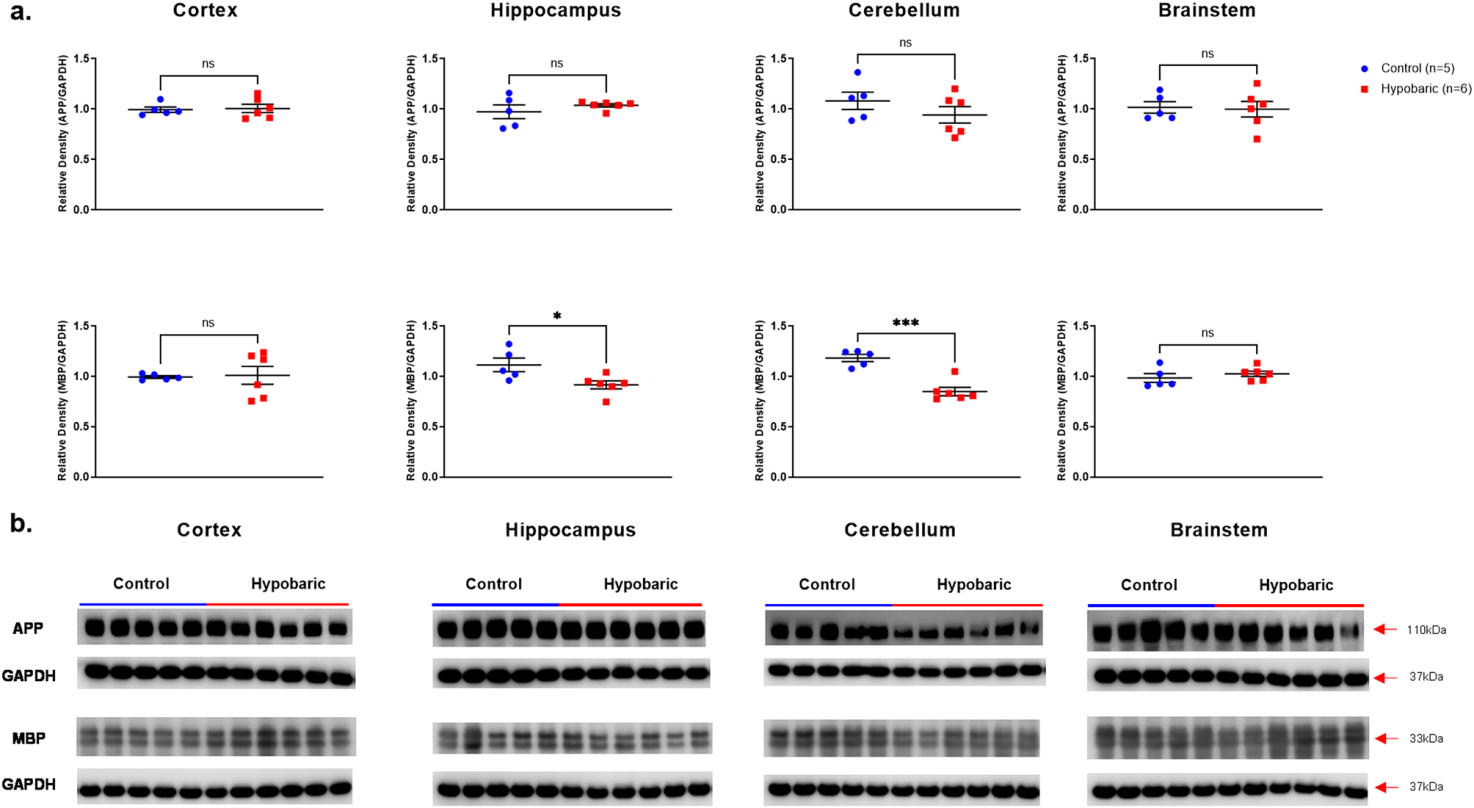
APP and MBP protein expression following hypobaric exposure. (**a**) Histograms representing the densitometric ratio of levels of APP and MBP with respect to GAPDH in the Control (blue spots) vs. HYPOBAR (red squares) group as measured in the frontal cortex, hippocampus, cerebellum and brainstem. All gels were run in duplicate and data represents the average of 2 runs per sample. Error bars represent standard error of the mean (SEM). *p < 0.05; **p < 0.01 (two-tailed unpaired *t*-test). (**b**) Representative western blots for antibodies used. Full length blots are shown in Supplementary Figs. 3–9.

No changes in APP expression levels were detected in any brain region examined in HYPOBAR vs. C group (Fig. 9).

#### MBP

Significantly decreased levels of MBP were detected in Hippo (p = 0.026) and CRB (p = 0.0002) in HYPOBAR vs. C (Fig. 9). This further finding confirmed the observed trends for decreased MBP expression levels across the same brain regions examined at the histological level.

## Discussion

The effects of HA exposure have been associated with various systemic, cognitive, neurological, and psychiatric abnormalities^1,36–38^. While the prevalent view regarding the principal pathophysiological mechanisms related to the brain effects caused by HA have been generally attributed to hypoxia, the search and identification for some more specific neuropathological effects and brain molecular changes associated with hypobaria have not historically received much attention. Systematic neuropathological assessments and molecular quantitative analyses (WB) of a rare set of large mammalian brains experimentally exposed to nonhypoxic-hypobaric vs. nonhypoxic-normobaric conditions allowed us to quantitatively measure some hypobaria-associated molecular changes. In particular, increased levels of soluble pTau (S202/CP13 and AT8) across different brain regions of HYPOBAR vs. C animals were found. Moreover, increased levels of soluble pTau were accompanied by the intracellular accumulation of insoluble forms of pTau (AT8) in a specific neuronal population localized at the layer III of the miniswine cortex.

We hypothesize that higher levels of soluble pTau, together with accumulated insoluble forms of it in more hypobaric-sensitive neurons across different brain regions as shown when comparing HYPOBAR vs. C animals, could represent a major and specific pathophysiological effect and a signal for a more global reacting brain process triggered mainly, if not exclusively, by hypobaric conditions. In fact, the possibility of measuring the molecular effects due to hypoxic instead of hypobaric conditions were accurately minimized, if not entirely eliminated, by the experimental design that was used in this investigation. Furthermore, the observed higher levels of pTau were associated with variable levels of total Tau (HT7) across the different brain regions examined. Such hyperphosphorylated-tau/total-Tau ratio changes are not actually novel and have been observed in several Tau-related neurodegenerative conditions^39,40^. Generally, hyperphosphorylated-Tau/total-Tau ratio changes are considered to be part of molecular pathomechanisms associated with early neuronal dysfunctions as well as with cognitive and behavioral clinical abnormalities affecting specific neuronal types^25,41–43^. Nonetheless, pTau/Tau ratio abnormalities could also be due to phenomena of Tau gene downregulation, which has recently been shown to be involved in neuronal outgrowth and myelin transport processes^44^. Although the physiological role of Tau gene downregulation is not fully understood, a series of investigations have shown that Tau protein (and its related gene regulation and alternative RNA splicing) is actually involved in fundamental neuroplasticity and axonal transport mechanisms that control some specific cytoarchitectural aspects of neurons and glial cells during brain development, normal adulthood, and normal aging^45,46^. Consequently, it would not be surprising if the hypobaria could stimulate neuroplasticity-tau related phenomena due to fundamental changes of the environmental working conditions for the brain.

In support of pTau-related changes, the stereology-based neuronal counting of AT8-positive neurons in the DG of the Hippo showed a higher number of AT8-positive neurons in the HYPOBAR vs. C group. This finding represents another new set of stereology-based quantitative data supporting a direct effect of the hypobaria on the brain, and Hippo in particular. Importantly, apart from the Hippo, also other brain regions showed accumulation of AT8+ neurons, except the CRB. This dissociation in terms of pTau accumulation between cerebral vs. cerebellar areas is not actually totally new. In fact, in AD for example, it is well known that pTau accumulation (i.e. neurofibrillary tangles [NFT]) is rarely observed in the CRB even in very advanced cases of AD pathology. This hypoxia-related dissociation between cerebrum and CRB in terms of pTau insoluble accumulation seems to reinforce the general concept about specific metabolic and pathophysiological mechanistic differences between cerebellar tissue and other regions of the CNS. Intriguingly, though, as our findings show, the region-based pTau accumulation dissociation between cerebral vs. cerebellar regions does not correspond to a similar dissociation in terms of neuroinflammatory response (either as for astroglia reactivity and microglia response). In fact, both histopathological and molecular GFAP and IBA1 changes were observed across all examined brain regions, including the cerebellum.

Altogether, these molecular and pathological findings suggest that the hypobaria, per se, is an important environmental parameter and possible risk factor for determining higher levels of soluble pTau and consequentially, abnormal accumulation of insoluble pTau in cerebrocortical neurons. As shown, hypobaria produces changes of the ratios between different forms of Tau, mainly between pTau S202 and AT8 vs. total-Tau (HT7). These changes can actually act in favor of the accumulation of insoluble pTau in some specific and more vulnerable neuronal types, which seem to include, among other, the hippocampal-DG neurons of large mammalian brains. Theoretically, these neuronal abnormalities in the Hippo could explain some of the memory-related issues observed in humans after exposure to normohypoxic-hypobaria conditions (e.g. U2-pilots at extreme-HA).

pTau neuronal accumulations appear to be accompanied by regionally-specific astroglial, microglial, and myelination abnormalities, which could further contribute to the manifestations of HA-related cognitive and non-cognitive symptoms generated by hypobaric conditions, even when hypoxia is avoided or minimized. Moreover, these new findings were supported by the observation that pTau AT8-positive neurons (as well as GFAP and IBA1 changes) were not observed in the brains of swine kept at SL with 21%FiO_2_ (C_SL_ group). However, it could be not totally excluded that an altitude of 5 K feet above SL (C group) or Hyperoxic conditions (FiO_2_ 100% in the HYPOBAR group) could have contributed to the observed pathological and molecular changes.

HA-hypobaria-related cortical pTau changes were associated with increased levels of GFAP (and astroglial morphometric changes) across almost all examined neuroanatomical regions, at different degrees. However, in terms of neuroinflammatory response, the CRB appeared to be one of the most affected brain regions, although not the only one. Here, it is important to emphasize that as neurons boast an impressive morphological and functional diversity across different cortical layers and brain circuits, glial cells have a similar, or even higher, level of morphological complexity and reactive diversity across different brain regions. Among the different types of glial cells identified, Bergmann glial cells (BGC) are considered a specific cerebellum-related type of astroglial cells. BGC are highly diversified astrocytes in comparison to astrocytes in the cerebrum, and they react in a quite different manner as well. Determining if HA, and specifically hypobaria, primarily affects cerebellar astroglial cells vs. other types of astroglial cells across different CNS regions was outside the scope of our analyses. However, these analyses will represent an important hypothesis to test in future studies. In support of this possible hypobaria-related cerebellar astroglial activation hypothesis though, it should also be recalled how ataxia and balance issues—clinical expressions of cerebellar dysfunction—are indeed clinical phenomena frequently reported as part of the acute mountain sickness syndrome^47^.

WB analyses showed some opposite directionality changes in terms of IBA-1 levels in HYPOBAR vs. C group. In fact, trends of lower IBA-1 levels in the Cortex and BS and trends of higher levels of IBA-1 in Hippo with a significant difference in the CRB, were observed. If confirmed by future larger studies, these additional findings related to a region-based IBA-1 differential reactivity could suggest that microglia reactivity is different, and even in an opposite direction, across various neurocircuits at a given time point for a specific pathological or reacting process. Moreover, IBA-1 levels and microglia reactivity in general, could be related to specific adaptive or neuroplasticity processes occurring in specific neuro-anatomo-functional regions and triggered by specific molecular inputs, metabolic changes, or environmental signals such as hypobaria.

Strikingly, at neurohistological and molecular levels, hypobaria appeared to affect myelination levels. Lower levels of MBP (and myelin rarefaction by LFB-PAS stain) were present in the Hippo and CRB (together with a trend of lower level in the Cortex). These findings acquire special relevance when considering recent investigations that showed a direct association between Tau loads and myelination loss, identified actually as WMH by MRI across different Tau-related neurodegenerative human conditions^48^. LFB-PAS qualitative evaluations confirmed myelin rarefaction phenomena across most of the WM areas subjacent to cortical gyri in HYPOBAR vs. C animals, without any specific mechanism of sparing or affecting U-fibers, which are considered, among other, as the slowest myelin fibers developing in the CNS and linked to the very advanced stages of brain development. These HA-related myelin changes represent the best “candidate” lesions in order to explain the WMH observed in U2-pilots. However, specific imaging-pathological investigations are needed to confirm this hypothesis.

These neuropathological and molecular findings in larger mammalian brains exposed to nonhypoxic-hypobaric conditions are novel and suggest that hypobaria is able to induce the intra-neuronal accumulation of insoluble pTau accompanied by astroglial and microglial reactions, together with reduced levels of myelination across subcortical areas. While direct cognitive-behavioral correlations were not possible in this study, these new findings support the hypothesis that hypobaric conditions represent a major risk factor for the development of brain lesions and triggering of pathogenetic mechanisms more typically associated with cognitive disorders such as MCI, AD, bvFTD, and other neurodegenerative conditions. In particular, AT8-positive lesions in the Hippo may be related to cognitive issues recorded in some pilots and climbers, which together with cerebellar alterations, also encompass a neuroinflammatory reaction as indicated in this study by the increased levels of GFAP and IBA-1 in the Hippo and CRB. However, if pTau lesion accumulation, myelin rarefaction phenomena, and neuroinflammatory response are directly linked to the WMH observed in U2-pilots is not completely clear yet and further imaging-pathological correlation and genetic studies are needed.

### Strengths, limitations and future directions

One of the major points of strength of these new findings consists in the fact that we were able to analyze brain tissues from larger mammalian brains (miniature swine brains), which are anatomically and physiologically much more similar to humans compared to rodents. In addition, we employed different methods of analyses (neurohistology, IHC, and WB) that mutually confirmed these new HA-hypobaria-related observations.

However, we also acknowledge the limitations of this study. In fact, the study did not include both sexes and animals at different ages, which could have generated more generalizable results and aging-related HA data as well. In fact, while the precise determinants of sex-differences in terms of HA-consequences on the brain are not known, it has been reported that males and females can react differently when exposed to the same HA parameters^49^. Whether these sex-related differential effects are due to genomic factors, different cerebrovascular responsiveness, physiological or hormonal factors, is not completely understood. Due to the initial nature of our neuropathological investigation, we recognize that a more detailed mechanistic explanation could not be provided at the moment. At hypothetical level though, we propose that anatomical barometric sensors, likely localized in the BS and/or CRB, could play a major pathophysiological role in the types of brain lesions and molecular changes observed in our investigation. Finally, we would also like to emphasize the technical difficulties in replicating these results since swine studies are quite expensive and require dedicated facilities and specialized personnel for their success.

In sum, our data support the hypothesis that cerebral and cerebellar expression level changes of specific brain molecules (pTau) involved in human cognitive disorders are generated as sequelae of hypobaric conditions and that both astroglial and microglial reactive phenomena are also present. It is still unknown, though, if these neuropathological and molecular changes represent only initial aspects of possible long-term consequences on cognition and behavior abnormalities for different occupational categories exposed to HA or extreme-HA, such as military pilots. Also, it remains to determine if genetic and other biological or environmental aspects, as well as preventive pre-flight/climbing procedures would be able to positively act against the apparent molecular-neurodegenerative process activated by HA, and hypobaria in particular. Finally, it would be also important and necessary to explore if compensatory or reparative neuroplasticity mechanisms do occur in response to HA in general, and hypobaria in particular.

## Methods

### Experimental design, animal features, and simulated high altitude exposure protocols

15 female pre-pubescent *Sinclair* miniature swine (*Sus scrofa domesticus*) were assessed under different environmental conditions to evaluate the impact of hypobaria. The experimental group consisted of swine that experienced simulated flight in altitude chambers to an altitude of 30,000 feet (30Kft) at 100% fraction of inspired oxygen (100%FiO_2_) for a total of 12 flights (altitude chambers), each lasting 8 h, occurring every 2 days over a span of 24 days. The second group of swine also underwent similar flight protocols (altitude chambers) but were part of the original cohort who were subjected to an altitude of 5000 feet (5Kft) at 21% FiO_2_ (21%FiO_2_) for total of 6 flights occurring every 3 days. The rationale for using 5Kft and 21%FiO_2_ as experimental conditions for the C group was based on the rationale to keep the experimental conditions as close as possible to real-life environmental testing conditions for U2-pilots (i.e. altitude of 5Kft, 21%FiO_2_, routine sinus check, sound of the chamber, constant temperature, etc.). Moreover, the flight frequency of one flight every 3 days mimicked the U2 pilots’ flight profile during peak military operations. Notably, 5Kft/21%FiO_2_ have been the testing conditions habitually used for pilots and other military personnel and under these conditions no hypoxia has been ever recorded on routine pulse oximetry. Moreover, brains from another age-matched group of animals that always lived at SL conditions that is, never been exposed to an altitude above SL and kept at 21%FiO_2_, were assessed in order to compare two control groups and exclude the possibility that even a HA of 5 K could generate detectable pathological consequences.

All animal protocols were reviewed and approved by the Institutional Animal Care and Use Committee, 59th Medical Wing, Joint Base San Antonio-Lackland, Air Force Base (AFB), Texas. All examined animals were miniature swine ranging between 90 and 95 days of age at the time of arrival at the Clinical Investigations & Research Support animal facility/vivarium, located at Lackland AFB. Specifically, the following experimental conditions were considered:

1. Sinclair miniature swine (*n* = 5, female) exposed to an altitude of 5000ft above SL (5Kft) with 21% of FiO_2_ (21%FiO_2_). This was the primary control animal group (C) for this experiment.
2. Sinclair miniature swine (*n* = 6, female) exposed to an altitude of 30,000ft above SL (30Kft) with 100%FiO_2_ (100%FiO_2_). This animal group was the nonhypoxic-hypobaric (HYPOBAR) group for this experiment. The 100%FiO_2_ was used to minimize hypoxia-induced effects during the experiment.
3. Sinclair miniature swine (*n* = 4, female) not exposed to HA (sham-animal). This additional third group of animals consisted of four *Sinclair* mini-swine who served as secondary sham control group of animals with 21%FiO_2_ and kept at SL. This group of animals was part of another experiment (Foreign Body study), which assessed the impact of small organic and inorganic foreign bodies in the bronchi on overall lung/bronchial parenchyma over a period of 3 weeks^50^. This animal group was termed Control-Sea Level group (C_SL_). For this C_SL_ group only formalin-fixed brain tissues were available, and no WB analyses were performed.

Following completion of the (simulated) flights, all swine were euthanized and the brains were fixed in 10% buffered formalin, or partially frozen, and sent to the DoD/USU Brain Tissue Repository and Neuropathology Program at USU, Bethesda, MD for neuropathological evaluation and molecular analyses (see “Neuropathology assessment”).

### Neuropathology assessment

At necropsy, after opening the skull, all brains were collected by dissecting the rostral portion of the cervical spinal cord from the brainstem. For both C and HYPOBAR animal groups, half-brain (left hemisphere) from each animal was immediately fixed in 10% neutral buffered formalin. The other half-brain (right hemisphere) was immediately frozen at − 80 °C. Fresh-frozen hemi-brains were later neuroanatomically dissected and samples from the Cortex (Cortex), Hippocampus (Hippo), Cerebellum (CRB), and Brainstem (BS) were taken.

### Neurohistologic and immunohistochemistry procedures

Each formalin-fixed hemi-brain was cut into a series of 4–5 mm thick coronal slabs and tissue samples were sub-dissected for processing to paraffin blocks using an automated tissue processor (ASP 6025, Leica Biosystems, Nussloch, Germany). Paraffin embedded tissue blocks were cut in a series of 20, 5-μm thick consecutive sections. The first three sections were selected for hematoxylin and eosin (H&E), Luxol fast blue-periodic acid-Schiff (LFB-PAS) and Cresyl Violet (CV) stains, while the remaining sections were retained for immunohistochemistry (IHC) procedures.

For each antibody, IHC procedures were performed using a Leica Bond III automated immunostainer with a diaminobenzidine chromogen detection system (DS9800, Leica Biosystems, Richmond, IL, USA). The following antibodies were employed: anti-hyperphosphorylated tau (pTau), targeting phosphorylated epitopes at residue Ser202, mouse monoclonal antibody, dilution 1:2000, epitope retrieval time 10 min; (this antibody is also known as CP13, and was kindly donated by the lab of Dr. Peter Davies, Albert Einstein College of Medicine, New York, NY); anti-hyperphosphorylated tau (pTau), targeting phosphorylated epitopes at residues Serine 202 and Threonine 205 (also known as AT8), mouse monoclonal antibody, dilution 1:2000 epitope retrieval time 10 min, (MN1020; Thermo-Fisher Scientific, Waltham, MA, USA); anti-glial fibrillary acidic protein (GFAP), mouse monoclonal antibody GA5, 1:250, with bond heat-induced epitope retrieval, epitope retrieval time 10 min (PA0026, Leica Biosystems, Wetzlar, Germany); anti-ionized calcium-binding adapter molecule 1 (IBA-1) rabbit polyclonal, dilution 1:100, epitope retrieval time 10 min, (Wako 016-20001; FUJIFILM Wako Pure Chemical Corporation, Osaka, Japan); anti-myelin basic protein (MBP) a marker of myelination, mouse monoclonal antibody, 1:100, (MBP101, ab62631, Abcam, Cambridge, MA, USA); and anti-amyloid precursor protein (APP), mouse monoclonal antibody clone 22c11, dilution 1:10, epitope retrieval time 10 min, (MAB348; EMD Millipore, Burlington, MA, USA).

All stained sections were digitally scanned using an Aperio scanner system (Aperio AT2—High Volume, Digital whole slide scanning scanner, Leica Biosystems, Inc., Richmond, IL, USA) and stored in a digital image archive for further assessment and analyses. Preliminary neuropathologic assessments for each section were performed using Aperio ImageScope (Aperio ImageScope, version 2016, Leica Biosystems, Inc., Richmond, IL, USA) to verify presence of immunoreactivity (IR), anatomical localization and neurohistological distribution of each antibody. After the preliminary ImageScope histological inspections (magnification at 20×), a Zeiss Imager A2 (ImagerA2 microscope, Zeiss, Munich, Germany) bright-field microscope with higher magnification lenses (40×, 63×-oil immersion objective) was used to identify and photograph histologic and pathologic details, as needed.

### Immunofluorescence procedures

Formalin-fixed paraffin embedded mounted sections were first deparaffinized in an oven at 60 °C for 45 min before being rinsed three times 10 min in Xylene, followed by two times 5 min rinses in decreasing alcohol concentrations of 100%, 95%, and 70%. Slides were then rinsed in water 5 min before an antigen retrieval step. Slides were placed in a 0.01 M citrate buffer composed of citric acid and trisodium citrate dehydrate at a pH of 6.0. They were heated in a microwave for five minutes and then allowed to cool at room temperature for 45 min. Slides were then rinsed in PBS three times 10 min. All tissue was labeled with phosphorylated tau, which recognizes tau phosphorylated at residues Serine 202 and Threonine 205 (AT8, 1:1000, MN1020, Thermo-Fisher Scientific, Waltham, MA USA) and co-labeled with either glial fibrillary acidic protein (GFAP; 1:500, 123895, Cell Signaling Danvers, MA, USA), ionized calcium binding adapter (Iba1; 1:100, ab178847, Abcam, Cambridge, MA, USA), or neuron-specific nuclear protein (NeuN; 1:500, 26975-1-AP, ProteinTech, Rosemont, IL, USA) and incubated overnight at 4 °C. Following incubation, slides were again rinsed in PBS three times 10 min and incubated for one hour in a solution of AlexaFluor secondaries for anti-mouse 594 (1:200, A11032, Invitrogen, Carlsbad, CA, USA) and anti-rabbit 488 (1:200, A11034, Invitrogen, Carlsbad, CA, USA). Sections were then rinsed and coverslipped using Vectasheild Vibrance with Dapi (H-1800, Vector Laboratories, Burlingame, CA, USA). Negative controls were done following the same protocol but omitting primary antibody. Fluorescence images were captured using an Allied Vision Pike camera (Statroda, Germany), attached to an Olympus VS120 microscope (Tokyo, Japan), and scanned at 20 X magnification.

### Protein extraction and western blot (WB) procedures

In order to compare the neuropathological observations obtained from formalin-fixed tissues from the left hemisphere (fixed-hemisphere) fresh*-*frozen (− 80 °C) brain tissues from corresponding right hemisphere (frozen-hemisphere) brain regions were warmed to − 20 °C in a cryostat. Each tissue sample was then specifically dissected into the following four anatomical regions: Cortex (Cortex; fronto-parietal cortex), Hippocampus (Hippo), Cerebellum (CRB), and Brainstem (BS). All samples/regions, which contained both gray matter (GM) and adjacent white matter (WM), were then homogenized in glass dounce homogenizers with ice cold lysis buffer (1 ml/100 mg tissue), which contained the following: 50 mM Tris–HCl (pH 8), 1% Igepal, 150 mM NaCl, 1 mM EDTA, 1 mM PMSF (protease inhibitor), 1 mM NaF (phosphatase inhibitor), and an additional protease inhibitor cocktail used at 1:100 concentration in the final lysis buffer mix (Sigma-Aldrich, P2714, St. Louis, MO, USA). Each sample was centrifuged at 12,000*g* for 20 min and supernatants collected, aliquoted and frozen at – 80 °C. Total protein content from each brain region (Cortex, Hippo, CRB, BS) was determined using the Micro BCA assay (Thermo-Fisher Scientific, 23235, Waltham, MA, USA). 20 µg of protein per sample, for all brain regions listed, were loaded on Novex Nupage 4–12% Bis–Tris Gels (Life Technologies, NP0329, Carlsbad, CA, USA) and were electrophoresed at 200 V constant for 30 min. Gels were transferred to PVDF membranes using the iBlot2 dry transfer method (Life Technologies, IB21001, Carlsbad, CA, USA). Membranes were blocked in 5% milk in 1× TBST for 1 h at room temperature (RT). The primary antibodies (see paragraph below) were diluted to the appropriate working concentrations in 5% milk in 1× TBST and incubated on the membranes overnight at 4 °C. Membranes were then rinsed 3 × 5 min in TBST. Appropriate HRP tagged secondary antibodies (see paragraph below) were diluted 1:2000 in 5% milk in 1× TBST and incubated on the membranes for 1 h at RT. Membranes were rinsed 3 × 5 min in TBST and 1 × 5 min in TBS. Membranes were incubated with chemiluminescent substrate (SuperSignal West Pico Chemiluminescent Substrate, Thermo-Fisher Scientific, 34577, Waltham, MA, USA) for 1 min and imaged on the LiCor C-Digit Blot Scanner (LiCor Biosciences, Lincoln, NE, USA). All membranes were stripped one time with Restore Plus Stripping Buffer (Thermo-Fisher Scientific, 46430, Waltham, MA, USA), for 10 min, rinsed with TBS and processed for immunoblotting as described above using GAPDH (1:40,000, 60004-1, Proteintech, Rosemont, IL, USA) for the loading control. Densitometry was performed with NIH ImageJ software (version 2.0.0) with all protein signal intensities normalized to GAPDH signal intensity.

#### Primary antibodies

To examine full length tau and phosphorylated tau expression levels the following antibodies were used: HT7, which recognizes all forms of Tau protein, (1:40,000, MN1000, Thermo-Fisher Scientific, Waltham, MA, USA); AT8, which recognizes tau phosphorylated at residues Serine 202 and Threonine 205 (1:500, MN1020, Thermo-Fisher Scientific, Waltham, MA USA) and pTau (S202) antibody (1:5000, ab108387, abcam, Cambridge, MA USA), which recognizes only the phosphorylated residue at Serine 202. To examine for possible diffuse axonal injury (DAI) we measured levels of amyloid precursor protein, APP-A4, (1:2000, MAB348, Millipore-Sigma, Burlington, MA, USA). To determine effects on proteins known to be associated with neuroinflammation, microglial activation, and demyelination, we measured levels of soluble glial fibrillary acidic protein (GFAP), a marker of astroglial cells (1:20,000, NCL-L-GFAP-GA5, Leica Biosystems, Newcastle Upon Tyne, UK); ionized calcium-binding adapter molecule 1 (IBA-1), a marker for microglial cells, (1:1000, ab178847, Abcam, Cambridge, MA, USA); and myelin basic protein (MBP), a marker of myelination, (1:10,000, ab7349, Abcam, Cambridge, MA, USA).

#### Secondary antibodies

The following HRP tagged secondary antibodies were used: Goat anti-mouse (1:2000 or 1:5000, ab97040, Abcam, Cambridge, MA, USA), Goat anti-rabbit (1:2000, ab97080, Abcam, Cambridge, MA, USA).

### Stereological measurements

Across all possible neuroanatomical regions of interest to perform cell counting comparisons between HYPOBAR vs. C, we focused on the Dentate Gyrus (DG) of the Hippocampus due to the well-defined anatomical localization, well-defined histological borders, and for the higher frequency, although not exclusive, of pTau-positive neurons in this region in HYPOBAR vs. C group. C_SL_ group was not included in the stereological assessment due to the complete negativity for pTau-positive neurons across all examined brain regions.

In order to quantify and compare between groups the number of Tau-positive lesions (intraneuronal accumulation of pTau [AT8]) stereological tools were used^51^. We estimated the number of pTau-positive cells in a total of four animals (*n* = 4) for each group (HYPOBAR and C). In fact, based on previous stereology studies and power analyses, a minimal amount of 4 randomly selected cases for each condition (with one of these conditions showing almost a total negativity to the lesion to measure [AT8-positive cells]) was the minimal sample size sufficient to detect significant differences. A single 10 µm-thick tissue section was randomly chosen, IHC stained with AT8 and was microscopically inspected at low (2.5×) and high (20×) magnification to assess presence and frequency of pTau lesions.

Measurements were performed using the Stereo-Investigator system, Version 11.0 (MBF Bioscience, Williston, VT, USA), equipped with a digital camera (AxioCam MRm, and MRc, Zeiss, Oberkochen, Germany) and multiple objective head (2.5×–63× oil-immersion). The sample grid area was 10,000 µm^2^, the counting frame dimensions were 40 × 40 µm, and the dissector height was 8 µm with guard zones of ± 1 µm^52^. The positive cell counting was performed using a 40× objective. A total of 8 cases were stereologically assessed, four randomly-chosen cases were taken from the HYPOBAR group and other four randomly-chosen cases were taken from the C group. The cell counting was performed on a single 10-μm thick randomly-chosen section dissected from the tissue block containing the hippocampus region and stained with AT8. All sections selected for stereological counting were randomly coded before assessment to perform the cell counting (DI) in a blinded manner.

### Statistics

For WB statistical analyses, a total of 11 samples (5 from the C and 6 from HYPOBAR group) and related quantitative data were available. For each antibody and for each examined region two-tailed unpaired *t*-tests were performed. Statistical significance for each measured protein expression level in each anatomical region examined was established when *p* ≤ 0.05. Statistical analyses and graph visualizations were performed using GraphPad Prism, Version 9.0, (GraphPad Software, San Diego, CA, USA).

As for stereological data, a two-tailed unpaired *t*-test between HYPOBAR and C group data was performed. After breaking the codes, for each case the total number of optical markers positive for intracellular accumulation of pTau (AT8 positive neurons) in DG neurons were included in the corresponding category for statistical analyses.

### Disclosure

The opinions expressed herein are those of the authors and not necessarily representative of those of the Uniformed Services University of the Health Sciences (USU), the Department of Defense (DOD), the United States Army, Navy, Air Force, VA, NIH, any other US federal agency, or of the Henry M. Jackson Foundation for the Advancement of Military Medicine (HJF), Inc.

### Ethics approval

All animal work was approved by the Institutional Animal Care and Use Committee at 59th Medical Wing, Joint Base San Antonio-Lackland, Air Force Base (AFB), Texas in compliance with the PHS Policy on Humane Care and Use of Laboratory Animals, the NIH Guide for the Care and Use of Laboratory Animals, and all applicable Federal regulations governing the protection of animals in research, as well as in compliance with the ARRIVE guidelines (https://arriveguidelines.org).

## Supplementary Information

Supplementary Legends.

Supplementary Figure 1.

Supplementary Figure 2.

Supplementary Figure 3.

Supplementary Figure 4.

Supplementary Figure 5.

Supplementary Figure 6.

Supplementary Figure 7.

Supplementary Figure 8.

Supplementary Figure 9.

## Data Availability

The datasets used and/or analyzed during the current study and supporting the conclusions of this article are included in this article. These datasets are also available from the corresponding author on reasonable request.
