# Supplementary material for: High altitude is associated with pTau deposition, neuroinflammation, and myelin loss
Source: Sci Rep. 2022 Apr 27;12:6839. doi: 10.1038/s41598-022-10881-x (PMC9046305; doi:10.1038/s41598-022-10881-x)
Supplement: Supplementary file 1 — Supplementary Legends. [file 41598_2022_10881_MOESM1_ESM.docx]

**Supplemental Figure 1.** Hematoxylin and Eosin stain across different left hemisphere brain regions in Control Swine vs. Hypobaric

**Supplemental Figure 2.** Cresyl Violet stain across different left hemisphere brain regions in Control Swine vs. Hypobaric

**Supplemental Figure 3**. Full length blots for AT8 and associated GAPDH. Blue bars identify the Control group and Red bars identify the Hypobaric group. The bands identified by green bars were from samples not used in this study. The bands under the green bars are not part of the analysis.

**Supplemental Figure 4.** Full length blots for pTau (S202) and associated GAPDH. Blue bars identify the Control group and Red bars identify the Hypobaric group. The bands identified by green bars were from samples not used in this study. The bands under the green bars are not part of the analysis.

**Supplemental Figure 5.** Full length blots for HT7 and associated GAPDH. Blue bars identify the Control group and Red bars identify the Hypobaric group. The bands identified by green bars were from samples not used in this study. The bands under the green bars are not part of the analysis.

**Supplemental Figure 6**. Full length blots for GFAP and associated GAPDH. Blue bars identify the Control group and Red bars identify the Hypobaric group. The bands identified by green bars were from samples not used in this study. The bands under the green bars are not part of the analysis.

**Supplemental Figure 7.** Full length blots for IBA1 and associated GAPDH. Blue bars identify the Control group and Red bars identify the Hypobaric group. The bands identified by green bars were from samples not used in this study. The bands under the green bars are not part of the analysis.

**Supplemental Figure 8.** Full length blots for APP and associated GAPDH. Blue bars identify the Control group and Red bars identify the Hypobaric group. The bands identified by green bars were from samples not used in this study. The bands under the green bars are not part of the analysis.

**Supplemental Figure 9**. Full length blots for MBP and associated GAPDH. Blue bars identify the Control group and Red bars identify the Hypobaric group. The bands identified by green bars were from samples not used in this study. The bands under the green bars are not part of the analysis.
