# Supplementary figures and images for: High altitude is associated with pTau deposition, neuroinflammation, and myelin loss
Source: Sci Rep. 2022 Apr 27;12:6839. doi: 10.1038/s41598-022-10881-x (PMC9046305; doi:10.1038/s41598-022-10881-x)

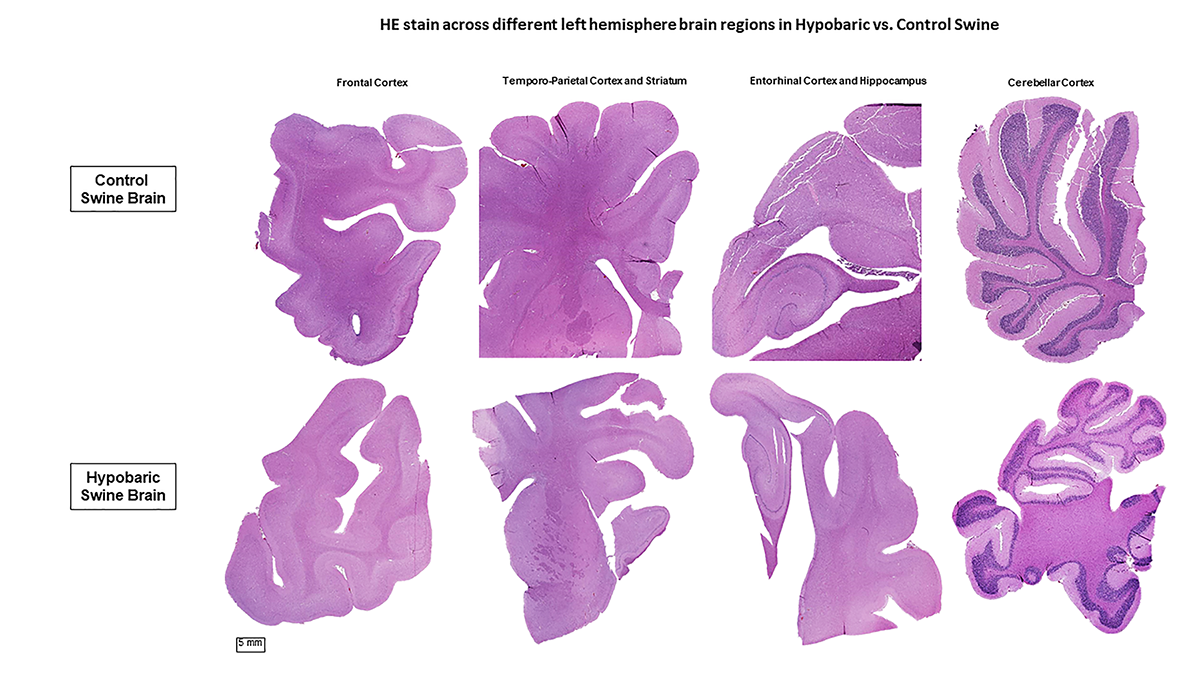

Supplement: Supplementary file 2 — Supplementary Figure 1. [file 41598_2022_10881_MOESM2_ESM.tif]

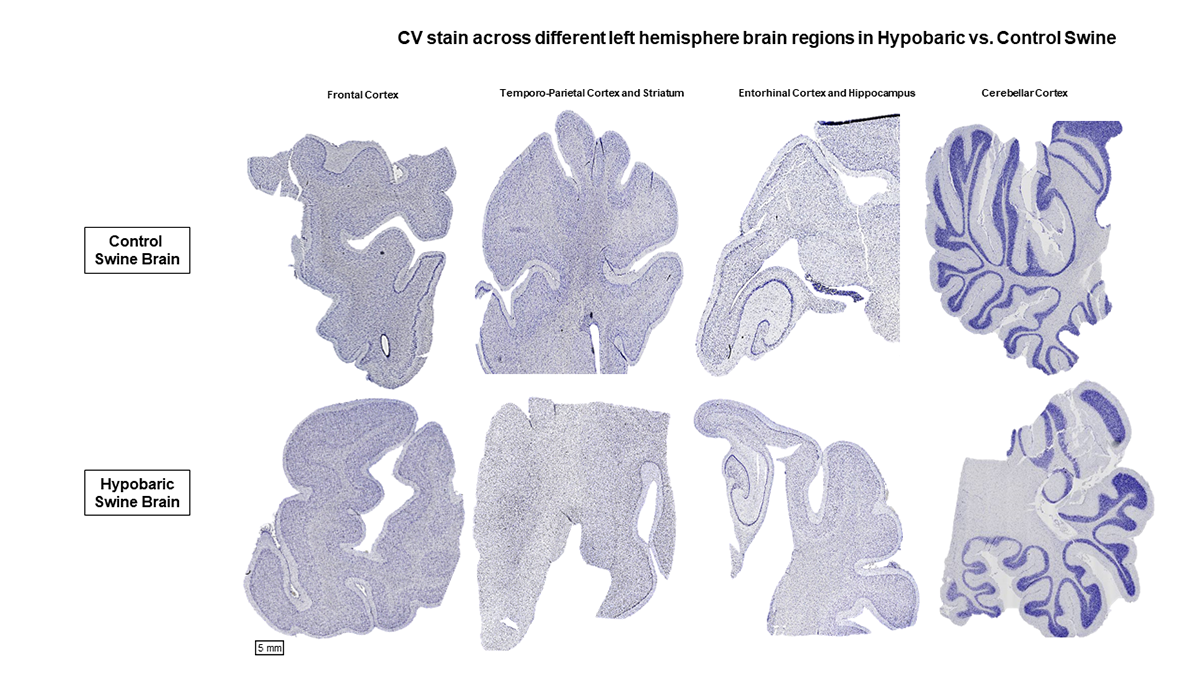

Supplement: Supplementary file 3 — Supplementary Figure 2. [file 41598_2022_10881_MOESM3_ESM.tif]

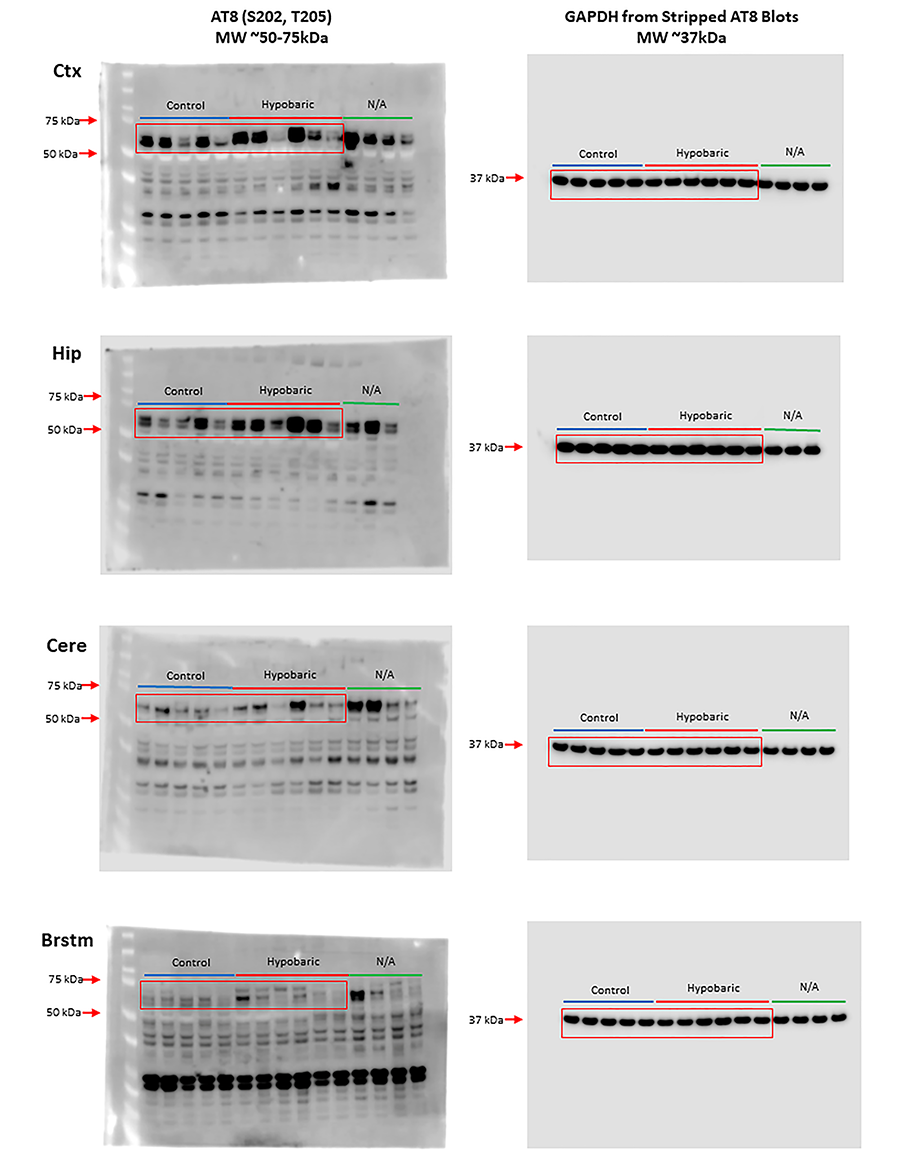

Supplement: Supplementary file 4 — Supplementary Figure 3. [file 41598_2022_10881_MOESM4_ESM.tif]

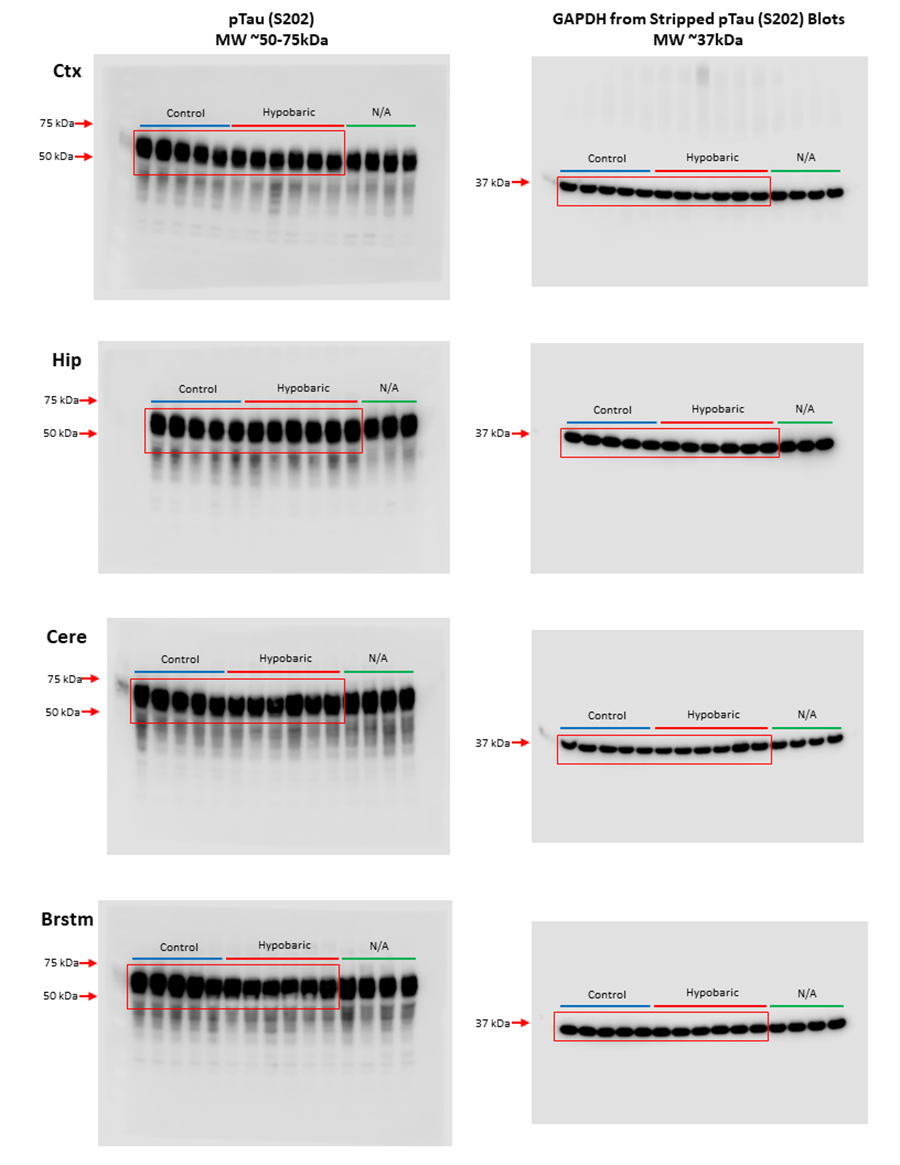

Supplement: Supplementary file 5 — Supplementary Figure 4. [file 41598_2022_10881_MOESM5_ESM.tif]

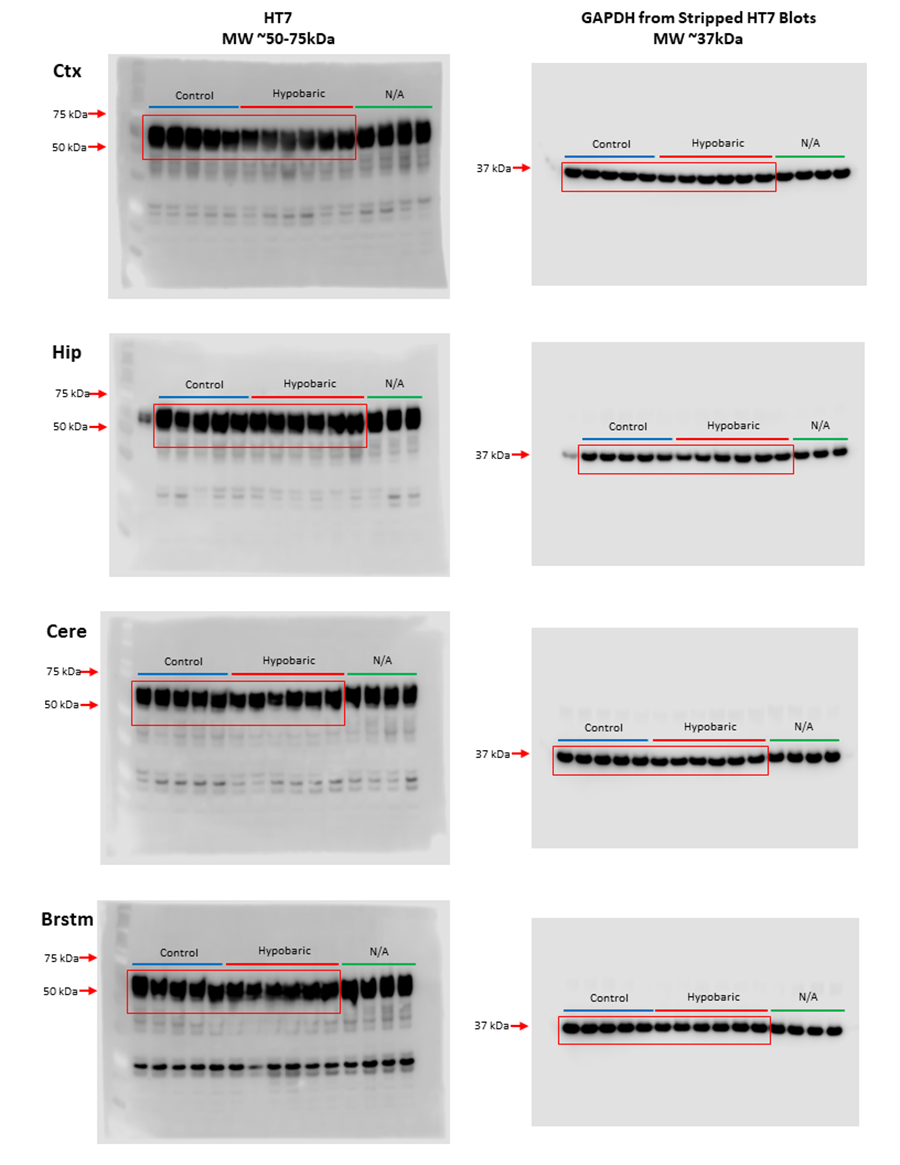

Supplement: Supplementary file 6 — Supplementary Figure 5. [file 41598_2022_10881_MOESM6_ESM.tif]

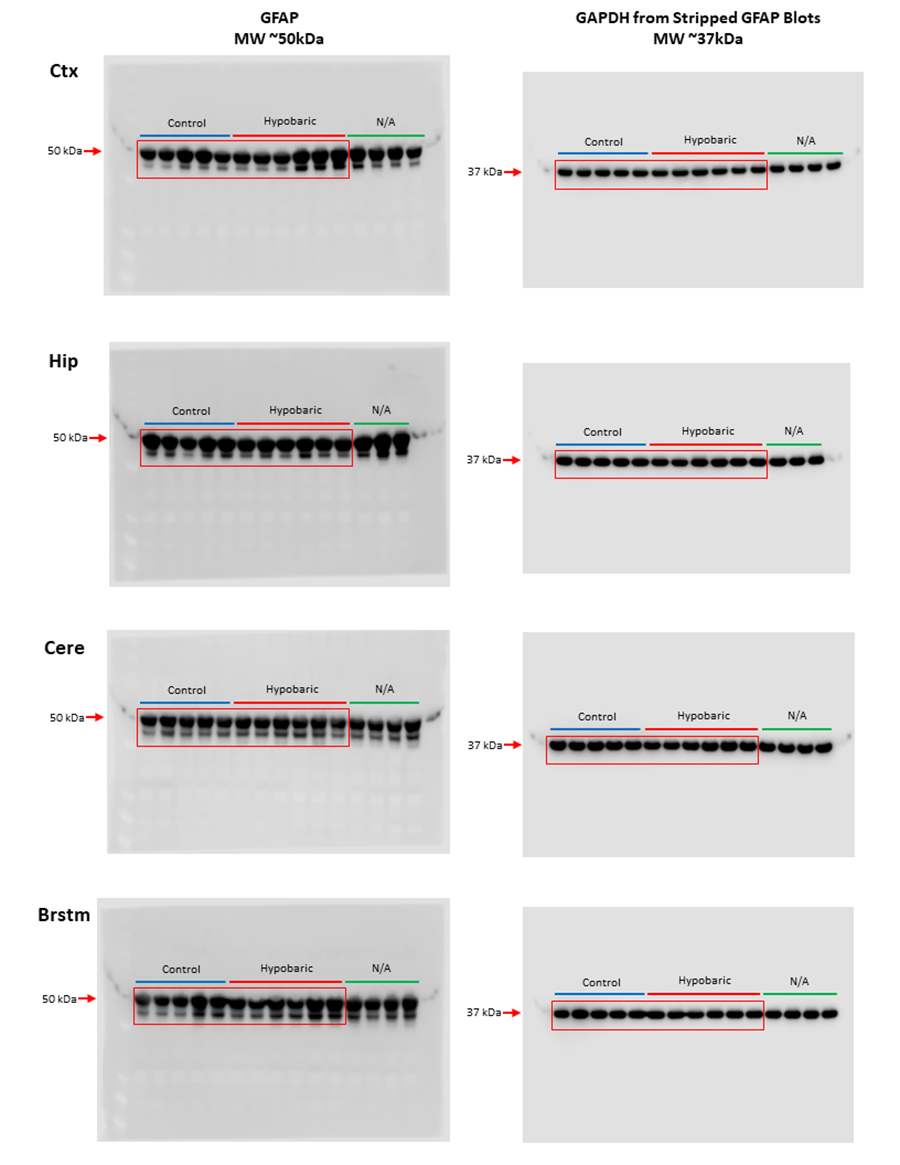

Supplement: Supplementary file 7 — Supplementary Figure 6. [file 41598_2022_10881_MOESM7_ESM.tif]

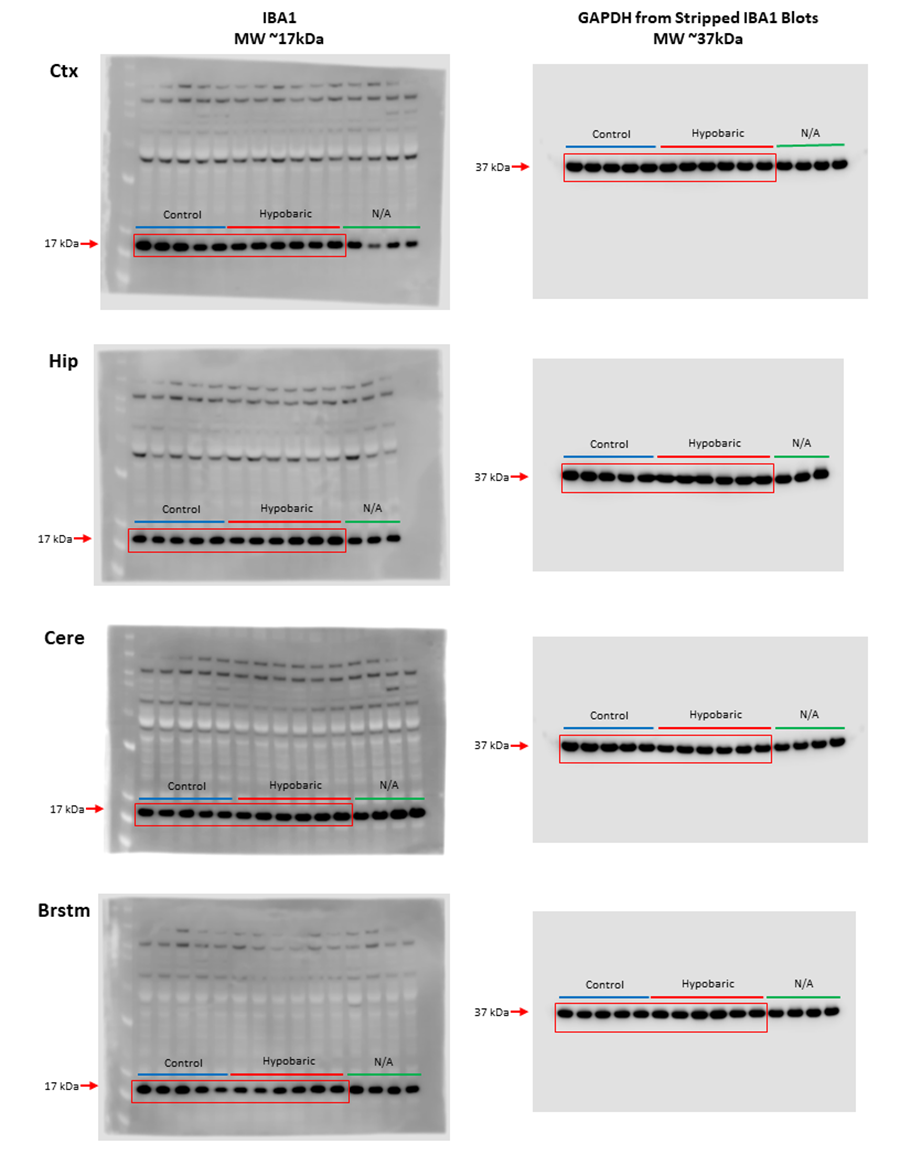

Supplement: Supplementary file 8 — Supplementary Figure 7. [file 41598_2022_10881_MOESM8_ESM.tif]

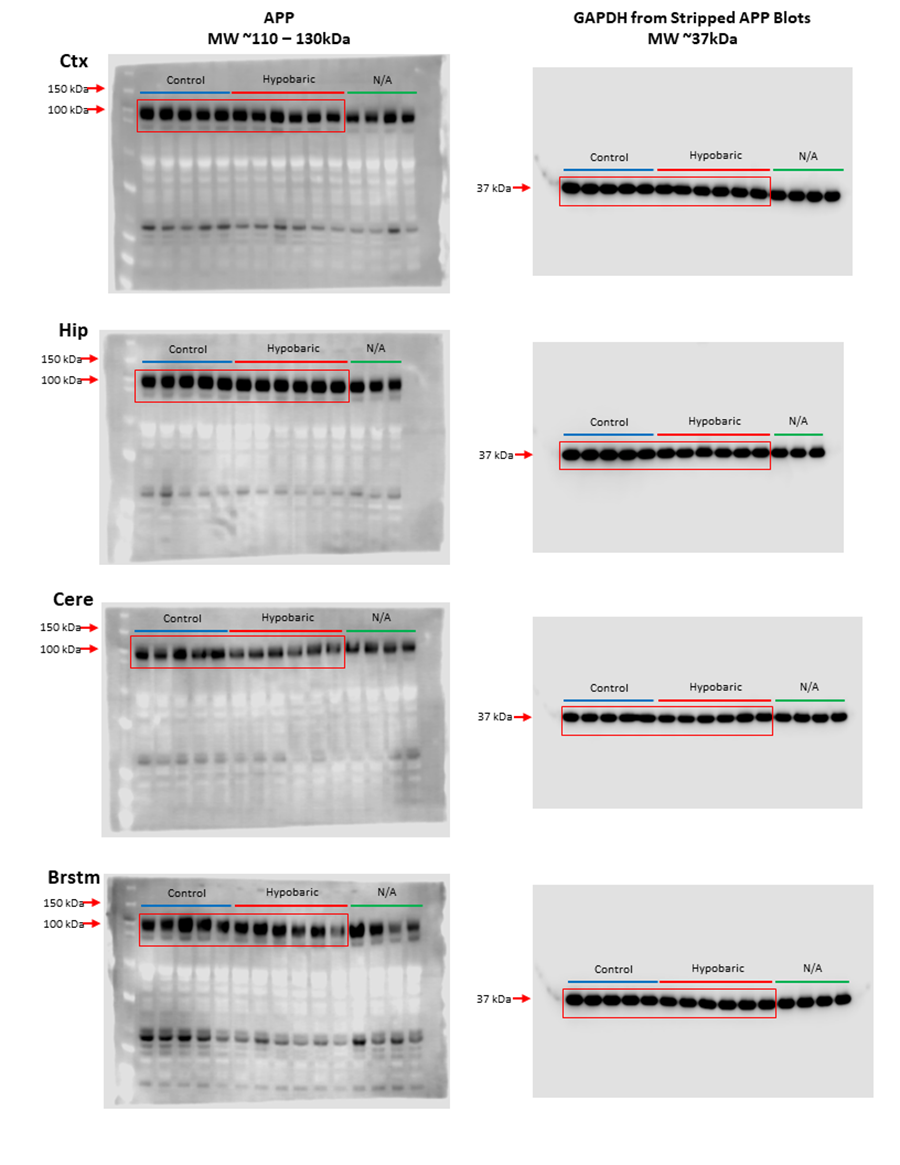

Supplement: Supplementary file 9 — Supplementary Figure 8. [file 41598_2022_10881_MOESM9_ESM.tif]

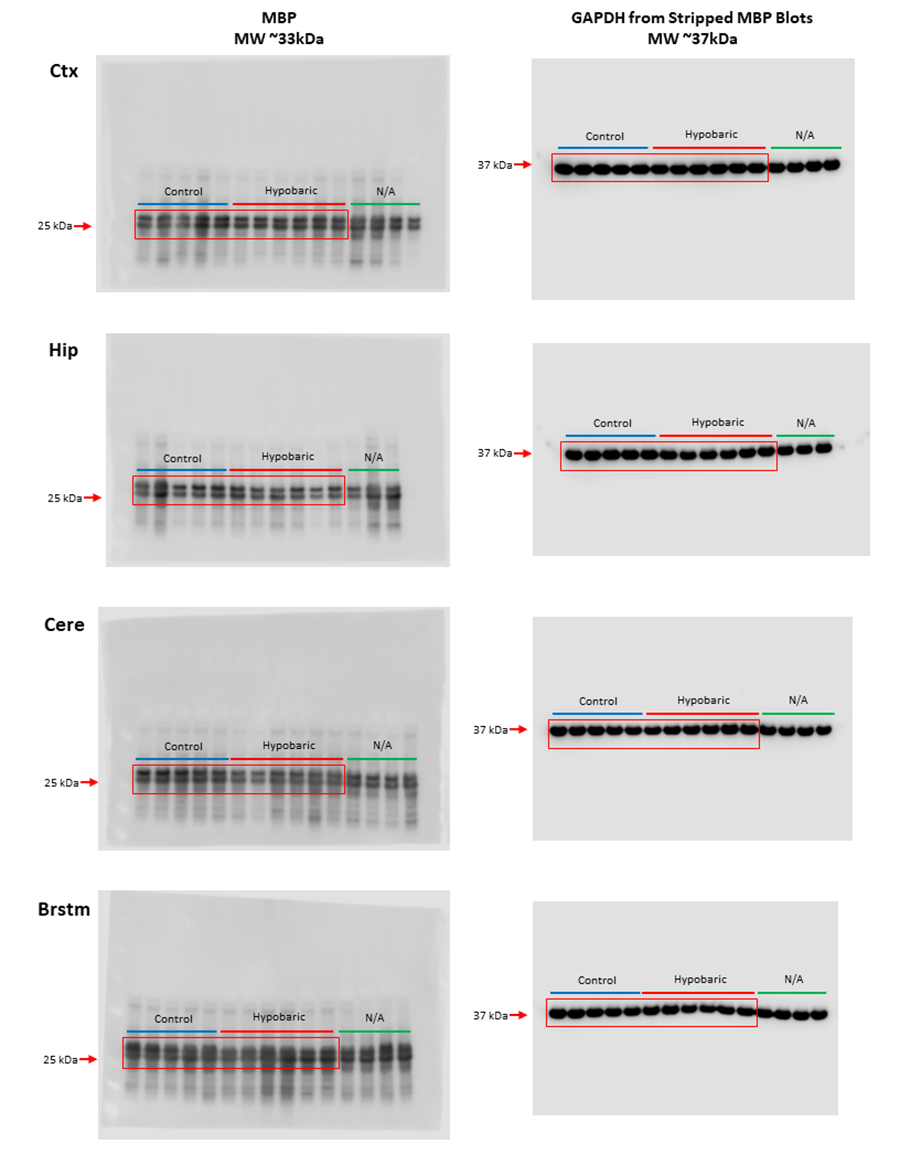

Supplement: Supplementary file 10 — Supplementary Figure 9. [file 41598_2022_10881_MOESM10_ESM.tif]
